# Combined effect of oxygen-scavenger packaging and UV-C radiation on shelf life of refrigerated tilapia (*Oreochromis niloticus*) fillets

**DOI:** 10.1038/s41598-020-61293-8

**Published:** 2020-03-06

**Authors:** Maria Lúcia Guerra Monteiro, Eliane Teixeira Mársico, Yhan da Silva Mutz, Vinicius Silva Castro, Rodrigo Vilela de Barros Pinto Moreira, Thiago da Silveira Álvares, Carlos Adam Conte-Junior

**Affiliations:** 1 Chemistry Institute, Universidade Federal do Rio de Janeiro (UFRJ), Rio de Janeiro, 21941-909 Brazil; 2 Center for Food Analysis (NAL-LADETEC), Rio de Janeiro, 21941-598 Brazil; 3 Department of Food Technology, Universidade Federal Fluminense (UFF), Rio de Janeiro, 24220-000 Brazil; 4 Nutrition Institute, Universidade Federal do Rio de Janeiro (UFRJ), Rio de Janeiro, 27979-000 Brazil; 5 National Institute of Health Quality Control, Fundação Oswaldo Cruz (FIOCRUZ), Rio de Janeiro, 21040-900 Brazil

**Keywords:** Biochemistry, Microbiology

## Abstract

This study investigated the physicochemical, instrumental and bacterial parameters of tilapia fillets subjected to oxygen-scavenger packaging, alone or in combination with UV-C radiation at two doses (0.102 and 0.301 J/cm^2^), stored at 4 ± 1 °C for 23 days. The oxygen scavenger, both UV-C doses, and the oxygen scavenger combined with UV-C, independently of the dose, extended the shelf life in 5, 6 and 7 days, respectively, by decreasing the bacterial growth rate and the formation of degradation compounds (e.g., TVB-N and ammonia). Oxygen-scavenger packaging, alone or in combination with UV-C at 0.102 J/cm^2^ and 0.301 J/cm^2^ showed lower amounts of free amino acids (FAA; 34.39, 34.49 and 34.50 mg L-lysine/kg fish tissue, 3.63, 3.57 and 3.61 mg L- ornithine/kg fish tissue, 27.52, 27.63 and 27.67 mg L-arginine/kg fish tissue), biogenic amines (BA; 3.81, 3.87 and 3.89 mg cadaverine/kg fish tissue, 12.88, 12.91 and 12.86 mg putrescine/kg fish tissue, 2.41, 2.44 and 2.47 mg spermidine/kg fish tissue), redness (2.53, 2.55 and 2.59), yellowness (6.65, 6.69 and 6.72), lipid oxidation (1.52, 1.53 and 1.58 mg malondialdehyde/kg fish tissue) and protein oxidation (5.06, 5.11 and 5.18 nmol carbonyls/mg protein), with higher hardness (3273.41, 2652.98 and 2687.57 g) than control (air packaging; 41.97 mg L-lysine/kg fish tissue, 4.83 mg L- ornithine/kg fish tissue, 37.33 mg L-arginine/kg fish tissue, 4.82 mg cadaverine/kg fish tissue, 16.56 mg putrescine/kg fish tissue, 3.21 mg spermidine/kg fish tissue, 4.26 of redness, 8.17 of yellowness, 2.88 mg malondialdehyde/kg fish tissue, 9.44 nmol carbonyls/mg protein and 2092.58 g of hardness), respectively, on day 13 of storage when the control fillets were unfit for consumption (7 log CFU/g) (p < 0.05). However, in the same day of storage, both UV-C doses had similar values for BA (p > 0.05), higher amounts of FAA (44.28 and 44.13 mg L-lysine/kg fish tissue, 5.16 and 5.12 mg L- ornithine/kg fish tissue, 40.20 and 40.28 mg L-arginine/kg fish tissue), redness (4.86 and 5.33), yellowness (9.32 and 10.01), lipid oxidation (3.09 and 3.52 mg malondialdehyde/kg fish tissue) and protein oxidation (10.27 and 11.93 nmol carbonyls/mg protein), as well as lower hardness (1877.54 and 1767.39 g), respectively, than control fillets (p < 0.05). The combined preservation methods were the most effective in extending the shelf life and prolonging the physicochemical quality of the refrigerated tilapia fillets and the O_2_ scavenger proved to be a potential alternative to prevent the negative changes induced by both UV-C doses.

## Introduction

Fish is rich in nutrients, but is highly perishable due to rapid endogenous enzyme and bacterial activity in the postmortem period, resulting in the production of undesirable metabolites (e.g., total volatile basic nitrogen, ammonia and biogenic amines), limited shelf life and loss of quality^1,2^. According to the United Nations Food and Agriculture Organization^3^, approximately 27% of the fish catch is discarded because of loss of quality between capture and final consumption, leading to economic loss. Nile tilapia (*Oreochromis niloticus*) is the most important freshwater fish species contributing to the increase in global production and consumption of fish from aquaculture systems^3^. Tilapia is usually consumed as fillets, which contain high amounts of protein (23%) and unsaturated fatty acids (66%), making the flesh more susceptible to protein and lipid oxidation^4,5^. Previous studies have been suggested a relationship between lipid and protein oxidation, wherein protein oxidation is favored by secondary compounds from lipid oxidation, and the free iron from protein oxidation catalyzes the lipid oxidation, causing changes in the color and texture and accelerating deterioration^4,6–9^.

Vacuum packaging (VP) and modified-atmosphere packaging (MAP) are widely used for fish flesh, to minimize oxygen-induced reactions and to inhibit the growth of obligate aerobic microorganisms; however, these packaging systems require costly equipment and do not prevent O_2_ from penetrating through the packaging film during storage^10,11^. O_2_ scavengers or O_2_ absorbers, which can prevent O_2_ penetration, are commercially available in the form of sachets, labels, cards or films. Their mechanism of action is based mainly on iron oxidation, wherein ferrous oxide (Fe^2+^) is converted to ferric oxide (Fe^3+^), reducing O_2_ levels in the package to less than 0.01%^10,12^. O_2_ scavengers do not require the use of equipment and, therefore it may be an efficient and economical alternative to the use of VP and MAP. Additionally, the effectiveness of the O_2_ scavengers in increasing shelf life and preventing oxidative processes in fish species have been described in the literature^13–15^.

UV-C radiation (wavelengths of 200–280 nm) is an emerging non-thermal technology that is effective in improving the bacterial quality and extending the shelf life of fish flesh through direct action on the microbial DNA, by formation of cross-linking between thymine and cytosine, and indirect action by water radiolysis, releasing free radicals^16,17^. This technology has several advantages, including ease of implementation, low cost and absence of toxic residues^17^. Previous studies confirmed that UV-C radiation is able to reduce the bacterial growth rate during refrigerated storage of fish species^1,18^. However, in general, the UV-C doses needed to significantly extend the shelf life may lead to the production of reactive oxygen species (ROS), which remove a hydrogen atom from a weak C-H bond, consequently initiating a free-radical chain reaction and intensifying the oxidative processes, changes in texture and color during refrigerated storage^1,19^. This effect depends mainly on the type and load of microorganisms present in the food matrix and the food composition^1,11,17^ and UV-C is therefore not necessarily dose-dependent^20^. Deprivation of oxygen in the package during storage could minimize the adverse effects of UV-C radiation.

The demand for a longer shelf life while maintaining the physicochemical characteristics without the use of chemical preservatives has increased, and represents one of the main challenges for the food industry and scientific community. The number of studies on combined preservation methods has increased^1,7,13,14^, but there are no reports about the use of an O_2_ scavenger in combination with UV-C radiation to treat any food matrix. Therefore, this study investigated the effect of an O_2_ scavenger and two different doses of UV-C radiation (0.102 and 0.301 J/cm^2^), alone or in combination, on the quality attributes of Nile tilapia fillets stored at 4 ± 1 °C for 23 days.

## Material and Methods

### Experimental design

Twenty-five kilograms of fresh tilapia (*Oreochromis niloticus*) fillets packed in low-density polyethylene bags were purchased from a local fish farm in Rio de Janeiro, Brazil (22°27′46″S 042°39′10″W). Fillets (111.24 g ± 7.18 g each) were transported in ice chests to the laboratory, where they were individually packed in nylon/polyethylene bags (15 cm width, 22 cm height, 80 µm thickness) with barrier properties of 66.31 cc/m^2^/day for O_2_ transmission rate (OTR) and 4.91 gm/m^2^/day for water-vapor transmission rate (WVTR) according to the information from the manufacturer (Gabrilina, São Paulo, Brazil). The fillets were randomly divided into six treatments according to packaging conditions (air or oxygen scavenger) and exposure to different UV-C doses (0.102 J/cm^2^ or 0.301 J/cm^2^). The treatments were AP (air packaging), OSP (oxygen-scavenger packaging), AUV1 (air packaging + UV-C at 0.102 J/cm^2^), OSUV1 (oxygen-scavenger packaging + UV-C at 0.102 J/cm^2^), AUV3 (air packaging + UV-C at 0.301 J/cm^2^) and OSUV3 (oxygen-scavenger packaging + UV-C at 0.301 J/cm^2^). After the O_2_ scavenger sachets were placed and the samples were radiated with UV-C, they were stored at 4 ± 1 °C and analyzed for total aerobic mesophilic count (TAMC), total aerobic psychrotrophic count (TAPC), *Enterobacteriaceae* count, free amino acids, biogenic amines, total volatile basic nitrogen (TVB-N), ammonia (NH_3_), lipid oxidation, protein oxidation, and instrumental color and texture parameters. The packaging headspace was 47.88 ± 1.20 mL in all treatments. AP was evaluated on days 0, 1, 2, 3, 4, 5, 6, 9, 11 and 13; and OSP, AUV1, OSUV1, AUV3 and OSUV3 were evaluated on days 0, 1, 2, 3, 4, 5, 6, 9, 11, 13, 15, 17, 19, 21, and 23. The criterion for determining the days of storage was based on a predictive primary model designed by Baranyi & Roberts^21^, using the DMFit program version 2.0 (Institute of Food Research, Norwich, UK), until the stationary phases of the bacterial groups (TAMC, TAPC, and *Enterobacteriaceae* count) were reached. All experiment was carried out in duplicate (n = 2).

### Oxygen scavenger system

In the OSP, OSUV1 and OSUV3 treatments, an oxygen-scavenger sachet was placed inside the package before sealing. The sachet used was the Ageless SS-50, with O_2_ absorption capacity of 50 mL (Mitsubishi Gas Chemical Co., Inc., Tokyo, Japan). This sachet reduces oxygen levels through spontaneous iron oxidation, converting ferrous oxide (Fe^2+^) to ferric oxide (Fe^3+^) in the presence of oxygen, resulting in an O_2_ concentration <0.01% according to information from the manufacturer (Mitsubishi Gas Chemical Co., Inc., Tokyo, Japan).

### UV-C radiation exposure

After packaging, AUV1, OSUV1, AUV3 and OSUV3 were subjected to UV-C radiation in an apparatus containing six 30-W lamps and six 55-W lamps (Osram HNS, OFR, Munich, Germany) designed by Lázaro *et al*.^20^. The samples were placed in the center of the UV-C apparatus at a distance of 14 cm from the lamps. The intensity levels were monitored with a UV radiometer (MRUR-203, Instrutherm Ltda., São Paulo, Brazil) wrapped with the same sample packaging, and the exposure times were measured every 5 s until the doses of 0.102 ± 0.001 J/cm^2^ for AUV1 and OSUV1, and 0.301 ± 0.001 J/cm^2^ for AUV3 and OSUV3 were reached. These doses were chosen due to its effectiveness in increasing shelf life while causing physicochemical changes in refrigerated tilapia fillets conforming previously reported by some authors^1,4,7^.

### Bacterial analysis

Serial dilutions were inoculated through the pour-plate technique into Petri dishes containing a plate-count agar (PCA, Merck, Darmstadt, Germany) for TAMC and TAPC, and Violet-Red-Bile-Glucose agar (VRBG-agar, Merck, Darmstadt, Germany) for *Enterobacteriaceae*, using a Spiral Plater (Eddy Jet 2, IUL Instruments, USA) mode E50. TAMC, TAPC and *Enterobacteriaceae* were enumerated in the electronic counter (Flash & Go, IUL instruments, USA) after incubation at 37 °C for 48 h, 10 °C for 7 days, and 35 °C for 24 h, respectively^22^. The results were expressed as log CFU/g fish tissue.

### Free amino acids analysis

L-lysine, L-ornithine and L-arginine were analyzed as described by Gatti *et al*.^23^ with modifications in the sample deproteinization step. In brief, 0.1 g of sample (tissue) was mixed with 1 mL of 1.5 M perchloric acid (v/v) to remove proteins. After 2 min at room temperature, 0.325 mL H_2_O and 0.5 mL potassium carbonate were added. The tubes were centrifuged at 10,000 × *g* for 2 min. The sample (50 μL) was mixed with 50 μL H_2_O and 40 μL of 2,5-dimethyl-1H-pyrrole-3,4-dicarbaldehyde (DPD) reagent solution (v/v) for 10 min. 360 μL of the mobile phase (0.05 M triethylammonium phosphate buffer) was added to the derivatized solution, which was immediately analyzed by HPLC. The HPLC device was equipped with an ACE C18 3-μm reversed-phase column (250 × 4.6 mm I.D.), a 5-μm Ascentis C18 reversed-phase guard column (20 × 4.6 mm I.D.) and an RF-10AXL photodiode array detector (SHIMADZU, Kyoto, Japan), monitoring the absorbance at 320 nm. The results were expressed as mg free amino acids/kg fish tissue.

### Biogenic amines analysis

Cadaverine, putrescine and spermidine were determined according to the method of Lázaro *et al*.^24^, using an HPLC (SHIMADZU, Kyoto, Japan) equipped with a CBM-20A controller composed of an LC-20AD pump, SPD-M20A diode-array detector, CTO-20A oven and SIL-20AC autosampler. The amines were separated using a Spherisorb ODS2 C18 column (15 × 0.46 cm I.D., 5 μm particle size) for the stationary phase, and an acetonitrile:water mixture (42:58, v/v) as the mobile phase, under isocratic conditions. The biogenic amines were detected at 198 nm, and the results were expressed as mg biogenic amines/kg fish tissue.

### Determination of total volatile basic nitrogen (TVB-N) and ammonia (NH_3_)

TVB-N was determined by Conway’s microdiffusion method^25^ and the results were expressed as mg TVB-N/100 g fish tissue. Ammonia was quantified by the colorimetric method, using a UV-1800 spectrophotometer (SHIMADZU, Kyoto, Japan) at 425 nm according to the protocol of Rodrigues *et al*.^11^. Results were expressed as µg NH_3_/g fish tissue, based on a standard curve (R^2^ = 0.996) constructed from seven different NH_3_ concentrations (1 to 15 µg NH_3_).

### Determination of lipid and protein oxidation

Lipid oxidation was evaluated by the thiobarbituric acid-reactive substances (TBARS) assay according to the method of Yin *et al*.^26^ adapted by Joseph *et al*.^27^. The absorbance values were read at 532 nm, using a UV-1800 spectrophotometer (SHIMADZU, Kyoto, Japan), and the results were expressed as mg malonaldehyde (MDA)/kg fish tissue from a standard curve (R^2^ = 0.999) constructed with eight different MDA concentrations (0.5 to 400 µmol). Protein oxidation was evaluated by the carbonyl content, following the method of Oliver *et al*.^28^ with modifications^29,30^. The absorbance values were measured at 280 nm (protein) and 370 nm (carbonyl) by a UV-1800 spectrophotometer (SHIMADZU, Kyoto, Japan), and the results were expressed as nmol carbonyls/mg protein. Protein content was determined by a standard curve (R^2^ = 0.999) constructed from five different concentrations of bovine serum albumin (0.1–1.0 mg), while the carbonyl content was calculated using an absorptivity coefficient for the protein hydrazones of 21.0/mM/cm.

### Instrumental color measurements

Lightness (L*), redness (a*) and yellowness (b*) values were measured with an illuminant A, 8 mm-diameter aperture, and 10° standard observer through a Minolta CM-600D portable spectrophotometer (Minolta Camera Co., Osaka, Japan). The color parameters were determined at four random locations on the surface of each fillet immediately after it was removed from the packaging^31^.

### Instrumental texture profile

The texture-profile analysis (TPA) was measured utilizing a TA.XTplus Texture Analyser (Stable Micro Systems, Surrey, UK) equipped with a cylindrical P/36 R probe. Each fillet was cut transversely into four pieces (2 × 2 × 2 cm^3^), which were compressed twice to 50% of their original height with the time of 5 s between the two compression cycles, and pre-test, test speed, and post-test of 1 mm/s following conditions established by Sun *et al*.^32^. The parameters determined were hardness, chewiness, cohesiveness, springiness, and resilience.

### Statistical analysis

The experiment was conducted in duplicate, using a fully randomized design (n = 2). A linear regression analysis was performed separately for each treatment to investigate the relationship between each physicochemical parameter and days of storage. The area under the curve (AUC), calculated by the trapezoidal method, was used to calculate the total amount of each physicochemical parameter produced during a time interval. To identify differences in the AUC among treatments (AP, OSP, AUV1, OSUV1, AUV3 and OSUV3), a one-way ANOVA was used. An additional post-hoc test with Tukey’s adjustment was performed. All analyses were performed with a 0.05 confidence level, using GraphPad Prism version 5.00 (GraphPad Software, San Diego, California, USA). The bacterial growth curves were obtained by the predictive primary model^21^ through the DMFit program version 2.0 (Institute of Food Research, Norwich, UK), and the differences among treatments regarding bacterial growth parameters (lag, log and stationary phases) were identified by one-way ANOVA with Tukey post-hoc test (p < 0.05).

## Results and Discussion

### Bacterial growth during storage

**Figure 1:**
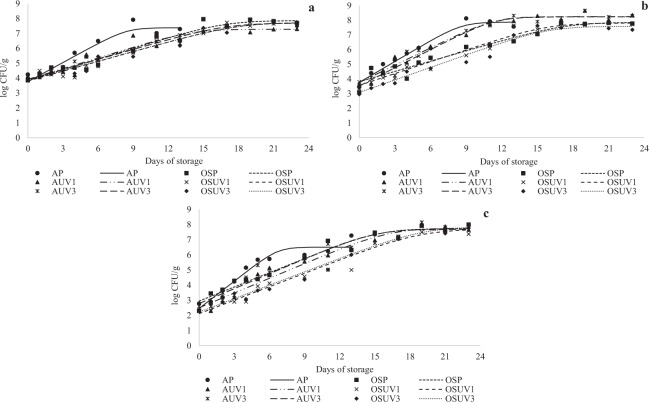
Total aerobic mesophilic count (**a**), Total aerobic psychrotrophic count (**b**), and *Enterobacteriaceae* count (**c**) in tilapia (*Oreochromis niloticus*) fillets non- and treated with oxygen scavenger packaging (OSP) and ultraviolet radiation (UV-C) stored at 4 ± 1 °C for 23 days. Results are expressed as the mean of log CFU (colony forming units)/g ± standard deviation (n = 2). AP (air packaging); OSP (oxygen scavenger packaging); AUV1 (air packaging + UV-C at 0.102 J/cm^2^); OSUV1 (oxygen scavenger + UV-C at 0.102 J/cm^2^); AUV3 (air packaging + UV-C at 0.301 J/cm^2^); and OSUV3 (oxygen scavenger packaging + UV-C at 0.301 J/cm^2^).

The results for TAMC, TAPC and *Enterobacteriaceae* are shown in Table 1 and Fig. 1a–c. The lag phase was absent in all bacterial groups. Although the number of colonies in the stationary phase of the fillets treated with the oxygen scavenger and/or UV-C radiation (0.102 or 0.301 J/cm^2^) was higher than in the fillets in air packaging (AP), these emerging techniques alone or in combination extended the shelf life of the tilapia fillets by decreasing (p < 0.05) the exponential growth rate (EGR) of the microorganisms (Table 1). The initial bacterial counts were 4.24 log CFU/g for TAMC, 3.45 log CFU/g for TAPC and 2.78 log CFU/g for *Enterobacteriaceae*. Considering the limit of 3 log CFU/g for initial counts of *Enterobacteriaceae* proposed by the International Commission on Microbiological Specifications for Foods^33^, the tilapia fillets showed good initial microbial quality. The limit of 7 log CFU/g for TAMC proposed by ICMSF^33^ was also used as the microbiological criterion to establish the shelf life of tilapia fillets during refrigerated storage. AP exceeded the limit of 7.0 log CFU/g for TAMC on day 9, while OSP, AUV1, AUV3, OSUV1 and OSUV3 reached this limit on storage days 14, 15, 15, 16 and 16, respectively.

**Table 1:** Bacterial growth parameters of tilapia (*Oreochromis niloticus*) fillets non- and treated with oxygen scavenger packaging (OSP) and ultraviolet radiation (UV-C) stored at 4 ± 1 °C for 23 days.

Microorganisms^¥^	Parameters^#^	Treatments^€^					
		AP	OSP	AUV1	OSUV1	AUV3	OSUV3
TAMC	Lag	0.00 ± 0.00	0.00 ± 0.00	0.00 ± 0.00	0.00 ± 0.00	0.00 ± 0.00	0.00 ± 0.00
	EGR	0.45 ± 0.01^a^	0.24 ± 0.01^b^	0.21 ± 0.01^b^	0.12 ± 0.01^c^	0.20 ± 0.01^b^	0.13 ± 0.01^c^
	NC	7.35 ± 0.01^b^	7.86 ± 0.02^a^	7.54 ± 0.34^ab^	7.72 ± 0.02^a^	7.70 ± 0.00^ab^	7.74 ± 0.02^a^
TAPC	Lag	0.00 ± 0.00	0.00 ± 0.00	0.00 ± 0.00	0.00 ± 0.00	0.00 ± 0.00	0.00 ± 0.00
	EGR	0.48 ± 0.01^a^	0.30 ± 0.01^c^	0.38 ± 0.01^b^	0.24 ± 0.01^d^	0.37 ± 0.01^b^	0.23 ± 0.01^d^
	NC	7.87 ± 0.14^b^	7.85 ± 0.00^b^	8.24 ± 0.01^a^	7.80 ± 0.02^c^	8.22 ± 0.02^a^	7.63 ± 0.04^d^
*Enterobacteriaceae*	Lag	0.00 ± 0.00	0.00 ± 0.00	0.00 ± 0.00	0.00 ± 0.00	0.00 ± 0.00	0.00 ± 0.00
	EGR	0.60 ± 0.00^a^	0.30 ± 0.01^b^	0.31 ± 0.01^b^	0.23 ± 0.00^c^	0.30 ± 0.01^b^	0.24 ± 0.01^c^
	NC	6.63 ± 0.17^b^	7.76 ± 0.04^a^	7.80 ± 0.06^a^	7.70 ± 0.01^a^	7.72 ± 0.04^a^	7.84 ± 0.05^a^

Results are expressed as means ± standard deviation (n = 2). ^a,b,c,d^Different letters in the same row indicate significant differences (p < 0.05) among treatments. ^¥^TAMC - Total aerobic mesophilic count; TAPC - Total aerobic psychrotrophic count. ^#^Lag – lag phase (h); EGR – exponential growth rate (log CFU/g/h); NC – number of colonies in the stationary phase (log CFU/g). ^€^AP (air packaging); OSP (oxygen scavenger packaging); AUV1 (air packaging + UV-C at 0.102 J/cm^2^); OSUV1 (oxygen scavenger packaging + UV-C at 0.102 J/cm^2^); AUV3 (air packaging + UV-C at 0.301 J/cm^2^); and OSUV3 (oxygen scavenger packaging + UV-C at 0.301 J/cm^2^).

The microbiota of tropical freshwater fishes such as tilapia is composed predominantly of Gram-negative aerobic and facultative anaerobic bacteria, including bacteria from the family *Enterobacteriaceae*, and Gram-positive bacteria^34^. Molinari *et al*.^35^ and Pakingking *et al*.^36^, evaluating the microbiota of tilapia, found a wide variety of bacterial genera and species, including *Pseudomonas* spp., *Shewanella putrefaciens*, *Aeromonas* spp., *Pasteurella pneumotropica*, *Photobacterium damselae*, *Plesiomonas shigelloides*, *Vibrio* spp., *Burkholderia cepacia*, *Chromobacterium violaceum*, and *Flavimonas oryzihabitans* (Gram-negative aerobic and facultative anaerobic bacteria); *Citrobacter* spp., *Edwardsiella* spp., *Enterobacter cloacae*, *Klebsiella oxytoca*, *Escherichia coli* (*Enterobacteriaceae*); and *Bacillus* sp. and *Staphyloccocus* sp. (Gram-positive bacteria). However, along with the increase in the storage time under aerobic conditions, *Pseudomonas* spp. became the dominant spoilage bacteria in refrigerated fish, due to low temperature^34^.

In our study, the oxygen scavenger and both UV-C doses (OSP, AUV1 and AUV3) had similar effects on EGR for TAMC and *Enterobacteriaceae*. However, UV-C radiation (AUV1 and AUV3) showed a higher (p < 0.05) EGR for TAPC than OSP. This fact may be explained by antimicrobial effect of the UV-C^16,17^. Gram-negative bacteria are more sensitive to UV-C radiation due to their lack of a thick cell wall, which prevents UV-C absorption by microbial DNA^37^. Nevertheless, although *Pseudomonas* spp. are Gram-negative, they are resistant to radiation due to their ability to form a biofilm in response to UV-C induced stress, in an attempt to repair damaged DNA^38,39^. On the other hand, obligate aerobic bacteria such as *Pseudomonas* spp. are highly sensitive to low oxygen concentrations from O_2_ scavenger^13^. Our results demonstrated that the O_2_ scavenger delayed the EGR of *Enterobacteriaceae*, which are facultative anaerobic bacteria. This delay can be attributed to the sensitivity of these bacteria to carbon dioxide, which increases in the package headspace due to the relative decrease in the O_2_ level caused by O_2_ scavengers^13,40^.

With respect to the combined preservation methods, the oxygen scavenger plus UV-C radiation, at both doses (OSUV1 and OSUV3), was the most effective in delaying the EGR in all bacterial groups, indicating a synergistic effect between the two preservation methods. While the O_2_ scavenger inhibits the growth of obligate aerobic bacteria and decreases the growth rate of facultative anaerobic bacteria of family *Enterobacteriaceae* by removing O_2_ and increasing the CO_2_ level inside the package, UV-C radiation decreases the growth rate of the microorganisms, especially Gram-negative bacteria, through direct or indirect damage to microbial DNA^13,16,17^.

OSP, AUV1, OSUV1, AUV3 and OSUV3 showed more viable cells in the stationary phase than AP. This difference may be explained by sublethal injury induced by CO_2_ and UV-C radiation to bacterial cells, which at first grow more slowly than intact cells, and more rapidly after recovery, mainly in an environment without natural competition^17,41,42^.

In agreement with the present results, previous researchers demonstrated that O_2_ scavengers were effective in extending the shelf life of refrigerated rainbow-trout fillets^14^ and ground beef^43^ by 5 and 2 days, respectively. Mohan *et al*.^15^ found an extension of 6–7 days in the shelf life of sardines packed with an O_2_ scavenger. Likewise, Bottino *et al*.^18^ reported that UV-C at 0.055 and 0.160 J/cm^2^ extended the shelf life of tambacu (*Colossoma macropomum* × *Piaractus mesopotamicus*) fillets stored at 4 °C by 50% and 100%, respectively. Monteiro *et al*.^1^ observed that the shelf life of refrigerated tilapia fillets exposed to UV-C radiation at 0.103 J/cm^2^ was extended by at least 2.5-fold.

### Free amino acids and biogenic amines

The levels of free amino acids (L-lysine, L-ornithine, L-arginine) and biogenic amines (cadaverine, putrescine, spermidine) increased in all treatments throughout the storage period (p < 0.05; Table 2). AUV1 and AUV3 showed higher total amounts (p < 0.05), while OSP, OSUV1 and OSUV3 had lower (p < 0.05) total amounts of free amino acids than AP throughout the storage period (Table 2). The results of free amino acids and biogenic amines in all days of storage can be found as Supplementary Table S1. The increase of free amino acids during storage is attributed to the action of endogenous and microbial proteolytic enzymes^44^. Our results are attributable to the resistance of *Pseudomonas* spp. to UV-C radiation, together with the effect of UV-C in increasing the amount of oxidized proteins, which are more susceptible to proteolysis, resulting in a high level of free amino acids^17,38,39^. On the other hand, oxygen scavenger is highly effective against *Pseudomonas* spp.^13^ and it is able to minimize ROS-induced oxidation^45^.

**Table 2:** Free amino acids and biogenic amines of tilapia (*Oreochromis niloticus*) fillets non- and treated with oxygen scavenger packaging (OSP) and ultraviolet radiation (UV-C) stored at 4 ± 1 °C for 23 days.

Parameters	Treatments^€^	AUC^¥^		Linear regression coefficients			
		AUC^¥^_0–13_	AUC^¥^_15–23_	y-intercept	slope	p-value	r-squared
L-lysine (mg lysine/kg fish tissue)	AP	367.30 ± 1.48^b^	NA	11.83 ± 0.79	2.49 ± 0.12	<0.0001	0.962
	OSP	302.40 ± 2.59^c^	324.20 ± 1.52^b^	11.72 ± 0.69	1.57 ± 0.06	<0.0001	0.966
	AUV1	402.90 ± 2.50^a^	396.20 ± 1.62^a^	17.76 ± 1.15	1.75 ± 0.09	<0.0001	0.927
	OSUV1	303.70 ± 1.30^c^	323.90 ± 0.79^b^	12.15 ± 0.63	1.54 ± 0.05	<0.0001	0.971
	AUV3	401.30 ± 1.08^a^	397.60 ± 0.61^a^	17.52 ± 1.13	1.77 ± 0.09	<0.0001	0.930
	OSUV3	304.20 ± 1.51^c^	324.20 ± 0.58^b^	12.16 ± 0.63	1.55 ± 0.05	<0.0001	0.970
L-ornithine (mg ornitine/kg fish tissue)	AP	47.04 ± 0.28^b^	NA	1.89 ± 0.14	0.26 ± 0.02	<0.0001	0.903
	OSP	34.15 ± 0.32^c^	33.56 ± 0.30^b^	1.66 ± 0.06	0.14 ± 0.01	<0.0001	0.962
	AUV1	51.62 ± 0.27^a^	47.90 ± 0.26^a^	2.60 ± 0.12	0.18 ± 0.01	<0.0001	0.926
	OSUV1	34.83 ± 0.44^c^	33.77 ± 0.38^b^	1.81 ± 0.05	0.13 ± 0.00	<0.0001	0.970
	AUV3	51.58 ± 0.39^a^	48.11 ± 0.34^a^	2.60 ± 0.12	0.19 ± 0.01	<0.0001	0.929
	OSUV3	35.08 ± 0.23^c^	33.82 ± 0.35^b^	1.82 ± 0.05	0.13 ± 0.00	<0.0001	0.968
L-arginine (mg arginine/kg fish tissue)	AP	337.80 ± 4.96^b^	NA	10.30 ± 1.09	2.34 ± 0.16	<0.0001	0.922
	OSP	244.10 ± 3.35^c^	275.00 ± 4.50^b^	9.63 ± 0.51	1.31 ± 0.04	<0.0001	0.973
	AUV1	373.70 ± 2.21^a^	393.60 ± 2.85^a^	15.04 ± 1.16	1.84 ± 0.09	<0.0001	0.933
	OSUV1	244.20 ± 1.65^c^	274.30 ± 1.03^b^	9.68 ± 0.45	1.31 ± 0.04	<0.0001	0.979
	AUV3	374.40 ± 2.12^a^	392.40 ± 2.44^a^	15.15 ± 1.18	1.83 ± 0.10	<0.0001	0.929
	OSUV3	244.30 ± 1.24^c^	274.00 ± 1.15^b^	9.75 ± 0.44	1.30 ± 0.04	<0.0001	0.980
Cadaverine (mg cadaverine/kg fish tissue)	AP	46.97 ± 0.54^a^	NA	2.14 ± 0.09	0.22 ± 0.01	<0.0001	0.945
	OSP	36.83 ± 0.28^b^	34.40 ± 0.19^b^	1.98 ± 0.04	0.12 ± 0.00	<0.0001	0.979
	AUV1	47.19 ± 0.31^a^	44.02 ± 0.18^a^	2.39 ± 0.09	0.17 ± 0.01	<0.0001	0.955
	OSUV1	36.94 ± 0.30^b^	34.28 ± 0.26^b^	1.98 ± 0.05	0.12 ± 0.00	<0.0001	0.974
	AUV3	47.35 ± 0.32^a^	44.01 ± 0.25^a^	2.42 ± 0.09	0.17 ± 0.01	<0.0001	0.955
	OSUV3	37.13 ± 0.28^b^	34.40 ± 0.26^b^	1.98 ± 0.05	0.13 ± 0.00	<0.0001	0.973
Putrescine (mg putrescine/kg fish tissue)	AP	178.40 ± 0.37^a^	NA	12.13 ± 0.10	0.25 ± 0.02	<0.0001	0.939
	OSP	155.70 ± 1.40^b^	110.30 ± 1.32^b^	11.15 ± 0.11	0.14 ± 0.01	<0.0001	0.890
	AUV1	178.60 ± 0.52^a^	129.70 ± 0.53^a^	12.35 ± 0.11	0.21 ± 0.01	<0.0001	0.953
	OSUV1	156.30 ± 0.42^b^	110.80 ± 0.62^b^	11.24 ± 0.11	0.14 ± 0.01	<0.0001	0.889
	AUV3	179.20 ± 0.70^a^	129.90 ± 0.46^a^	12.41 ± 0.10	0.20 ± 0.01	<0.0001	0.958
	OSUV3	155.90 ± 0.62^b^	110.50 ± 0.27^b^	11.26 ± 0.13	0.13 ± 0.01	<0.0001	0.880
Spermidine (mg spermidine/kg fish tissue)	AP	26.36 ± 0.44^a^	NA	0.96 ± 0.04	0.17 ± 0.01	<0.0001	0.977
	OSP	20.92 ± 0.29^b^	26.32 ± 0.38^b^	0.81 ± 0.04	0.13 ± 0.00	<0.0001	0.984
	AUV1	26.58 ± 0.30^a^	31.75 ± 0.39^a^	1.03 ± 0.04	0.16 ± 0.00	<0.0001	0.990
	OSUV1	21.04 ± 0.29^b^	26.26 ± 0.28^b^	0.83 ± 0.04	0.13 ± 0.00	<0.0001	0.985
	AUV3	26.51 ± 0.28^a^	31.97 ± 0.23^a^	1.02 ± 0.04	0.16 ± 0.00	<0.0001	0.988
	OSUV3	21.09 ± 0.21^b^	26.30 ± 0.19^b^	0.83 ± 0.03	0.13 ± 0.00	<0.0001	0.988

Results are expressed as means ± standard deviation (n = 2). ^a,b,c^Different superscripts in the same column indicate significant differences (p < 0.05) among treatments. ^¥^AUC – Area under curve; AUC0–13 – from day 0 to 13 among treatments AP, OSP, AUV1, OSUV1, AUV3, and OSUV3; AUC15–23 – from day 15 to 23 among treatments OSP, AUV1, OSUV1, AUV3, and OSUV3. NA – Not applicable. ^€^AP (air packaging); OSP (oxygen scavenger packaging); AUV1 (air packaging + UV-C at 0.102 J/cm^2^); OSUV1 (oxygen scavenger packaging + UV-C at 0.102 J/cm^2^); AUV3 (air packaging + UV-C at 0.301 J/cm^2^); and OSUV3 (oxygen scavenger packaging + UV-C at 0.301 J/cm^2^).

Regarding biogenic amines, cadaverine, putrescine and spermidine are formed mainly by bacterial decarboxylation of precursor free amino acids such as L-lysine, L- ornithine and L-arginine, respectively^46^. Metabolization of L-arginine to L-ornithine is another pathway to formation of putrescine^46^, which explains the high amount of this amine in relation to others (cadaverine and spermidine). The present study found no difference (p > 0.05) in the total amounts of cadaverine, putrescine and spermidine among AP, AUV1 and AUV3; whereas OSP, OSUV1 and OSUV3 resulted in lower (p < 0.05) total amounts of these biogenic amines than the other treatments (Table 2). Although O_2_ OSP, AUV1 and AUV3 had similar effect in controlling the growth of *Enterobacteriaceae*, which is the main bacterial group associated with the formation of biogenic amines^47^, UV-C radiation may cause oxidative decarboxylation of amino acids by catalyzing the production of Fe^3+^ ^48,49^. On the other hand, O_2_ absorber has the capacity to minimize the oxidative reaction pathways by oxygen scavenging^45^, explaining our results for combined preservation methods (OSUV1 and OSUV3).

Currently, there is little information about the effect of O_2_ absorbers and UV-C radiation on the production of free amino acids and biogenic amines in fish species during refrigerated storage. Similarly to our results, an increase in the amount of free amino acids by UV-C has been previously reported in fish stored at 4 °C^11,19^. Likewise, Mohan *et al*.^50^ observed a delay in the formation of putrescine, cadaverine and spermidine by use of an O_2_ scavenger in seer fish (*Scomberomorus commerson*) stored under refrigeration. However, no effect on the formation of putrescine, cadaverine and spermidine by similar UV-C doses was reported in freshwater fish species during refrigerated storage^11,19^.

### Total volatile basic nitrogen (TVB-N) and ammonia (NH_3_)

The initial levels of TVB-N and NH_3_ were 10.08 ± 0.00 mg TVB-N/100 g and 7.66 ± 0.04 µg NH_3_/g fish tissue. As expected, the TVB-N and ammonia levels increased (p < 0.05) in all treatments during the storage period, with the highest increases in the tilapia fillets under aerobic packaging (AP; Table 3). However, no treatment exceeded the limit of 25 mg TVB-N/100 g established by the Commission of the European Community^51^ until the end of storage, indicating that N-TVB was not a good indicator of bacterial spoilage and quality loss in tilapia fillets stored under refrigeration. On days 9, 14, 15, 16, 15 and 16 of refrigerated storage, when the acceptable microbial limit of 7 log CFU/g was reached, the TVB-N levels were 17.75 ± 0.89, 15.85 ± 0.07, 17.35 ± 0.81, 14.15 ± 0.27, 17.39 ± 0.10 and 14.09 ± 0.10 mg TVB-N/100 g for AP, OSP, AUV1, OSUV1, AUV3 and OSUV3, respectively (Supplementary Table S2). In freshwater fish species, TVB-N values are related mainly to the ammonia concentration, due to absence or low level of trimethylamine oxide *in vivo*^2,52^. However, there is no limit for ammonia content in freshwater fish species. In the present study, at the point when the fillets were unfit for consumption (7 log CFU/g), the ammonia levels were 10.60 ± 0.03, 10.44 ± 0.32, 11.46 ± 0.05, 9.99 ± 0.22, 11.47 ± 0.03 and 10.04 ± 0.20 µg of NH_3_/g of fish tissue for AP, OSP, AUV1, OSUV1, AUV3 and OSUV3, respectively (Supplementary Table S2).

**Table 3:** Physicochemical parameters of tilapia (*Oreochromis niloticus*) fillets non- and treated with oxygen scavenger packaging (OSP) and ultraviolet radiation (UV-C) stored at 4 ± 1 °C for 23 days.

Parameters	Treatments^€^	AUC^¥^		Linear regression coefficients			
		AUC^¥^_0–13_	AUC^¥^_15–23_	y-intercept	slope	p-value	r-squared
Ammonia (µg NH_3_/g fish tissue)	AP	129.80 ± 0.33^a^	NA	7.44 ± 0.09	0.40 ± 0.01	<0.0001	0.981
	OSP	117.90 ± 0.22^c^	90.33 ± 0.22^b^	7.65 ± 0.08	0.20 ± 0.01	<0.0001	0.970
	AUV1	122.80 ± 0.33^b^	94.43 ± 0.20^a^	7.88 ± 0.11	0.21 ± 0.01	<0.0001	0.953
	OSUV1	109.70 ± 0.19^d^	84.01 ± 0.16^c^	7.40 ± 0.04	0.16 ± 0.00	<0.0001	0.991
	AUV3	122.90 ± 0.32^b^	94.47 ± 0.24^a^	7.88 ± 0.12	0.21 ± 0.01	<0.0001	0.949
	OSUV3	109.80 ± 0.35^d^	84.16 ± 0.21^c^	7.40 ± 0.04	0.16 ± 0.00	<0.0001	0.991
TVB-N (mg TVB-N/100 g fish tissue)	AP	216.50 ± 9.09^a^	NA	11.98 ± 0.60	0.72 ± 0.09	<0.0001	0.788
	OSP	178.50 ± 4.24^c^	152.60 ± 3.34^b^	10.60 ± 0.31	0.45 ± 0.02	<0.0001	0.923
	AUV1	192.00 ± 4.94^b^	170.80 ± 3.04^a^	11.11 ± 0.35	0.54 ± 0.03	<0.0001	0.930
	OSUV1	154.00 ± 7.20^d^	125.60 ± 3.76^c^	9.86 ± 0.20	0.31 ± 0.02	<0.0001	0.928
	AUV3	191.70 ± 3.31^b^	171.10 ± 1.27^a^	11.08 ± 0.34	0.54 ± 0.03	<0.0001	0.932
	OSUV3	154.20 ± 3.70^d^	125.70 ± 1.73^c^	9.87 ± 0.18	0.31 ± 0.01	<0.0001	0.942
Lipid oxidation (mg malonaldehyde/kg fish tissue)	AP	19.46 ± 0.20^c^	NA	0.03 ± 0.00	0.22 ± 0.00	<0.0001	0.963
	OSP	9.84 ± 0.09^d^	20.91 ± 0.12^c^	0.09 ± 0.01	0.14 ± 0.01	<0.0001	0.960
	AUV1	23.13 ± 0.18^b^	28.88 ± 0.14^b^	0.49 ± 0.01	0.17 ± 0.01	<0.0001	0.934
	OSUV1	10.11 ± 0.11^d^	21.05 ± 0.20^c^	0.07 ± 0.00	0.14 ± 0.01	<0.0001	0.960
	AUV3	27.99 ± 0.30^a^	32.70 ± 0.10^a^	0.76 ± 0.01	0.18 ± 0.01	<0.0001	0.928
	OSUV3	10.36 ± 0.10^d^	21.07 ± 0.29^c^	0.04 ± 0.00	0.14 ± 0.01	<0.0001	0.963
Protein oxidation (nmol carbonyl/mg protein)	AP	71.08 ± 1.67^c^	NA	1.68 ± 0.16	0.58 ± 0.02	<0.0001	0.971
	OSP	43.78 ± 1.36^d^	48.35 ± 0.83^c^	1.71 ± 0.12	0.23 ± 0.01	<0.0001	0.951
	AUV1	88.09 ± 1.67^b^	97.15 ± 2.35^b^	3.60 ± 0.26	0.46 ± 0.02	<0.0001	0.944
	OSUV1	44.04 ± 1.85^d^	48.78 ± 0.62^c^	1.74 ± 0.13	0.23 ± 0.01	<0.0001	0.948
	AUV3	98.12 ± 2.65^a^	117.20 ± 4.80^a^	3.76 ± 0.29	0.58 ± 0.02	<0.0001	0.956
	OSUV3	44.71 ± 2.55^d^	48.69 ± 1.10^c^	1.80 ± 0.14	0.23 ± 0.01	<0.0001	0.941

Results are expressed as means ± standard deviation (n = 2). ^a,b,c,d^Different superscripts in the same column indicate significant differences (p < 0.05) among treatments. ^¥^AUC – Area under curve; AUC0-13 – from day 0 to 13 among treatments AP, OSP, AUV1, OSUV1, AUV3, and OSUV3; AUC15-23 – from day 15 to 23 among treatments OSP, AUV1, OSUV1, AUV3, and OSUV3. NA – Not applicable. ^€^AP (air packaging); OSP (oxygen scavenger packaging); AUV1 (air packaging + UV-C at 0.102 J/cm^2^); OSUV1 (oxygen scavenger packaging + UV-C at 0.102 J/cm^2^); AUV3 (air packaging + UV-C at 0.301 J/cm^2^); and OSUV3 (oxygen scavenger packaging + UV-C at 0.301 J/cm^2^).

AP had the highest (p < 0.05) total amounts of TVB-N and ammonia produced during the storage period (AUC), followed by tilapia fillets exposed to UV-C radiation alone, at both doses (AUV1 and AUV3), OSP alone, and OSP and UV-C in combination (OSUV1 and OSUV3; Table 3). These results agree with those obtained for bacterial growth parameters (Table 1) and free amino acids (Table 2), which are important substrates for ammonia formation^34^. Similarly, Bottino *et al*.^18^ and Monteiro *et al*.^1^ observed that, although the initial formation of TVB-N and ammonia in freshwater fish flesh was increased by UV-C radiation, it was still delayed during the storage period as a whole, compared to their control counterparts. The effectiveness of an O_2_ scavenger in reducing TVB-N and ammonia levels throughout refrigerated storage of fish species was also previously reported^13–15,45^.

### Lipid and protein oxidation

An increase in the malonaldehyde (MDA) and carbonyl levels was observed during refrigerated storage in all treatments, especially in AUV1 and AUV3 (Table 3). The increases in lipid and protein oxidation by UV-C radiation were dose-dependent. AUV3 showed the highest (p < 0.05) MDA and carbonyl levels during the storage period, followed by AUV1, AP, and treatments with the O_2_ scavenger (OSP, OSUV1 and OSUV3), which did not differ from each other (p > 0.05; Table 3).

A concomitant lipid and protein oxidation has been described in literature^6,9^ and it was also observed in our study. Lipid and protein oxidation occur mainly in the presence of reactive oxygen species (ROS)^6,53^. Therefore, our findings may be attributed to pro-oxidant properties of the UV-C radiation^6,17,54^ and capacity of the O_2_ scavenger in minimizing ROS-induced oxidation^45^. In agreement with the results of this study, an increase in the MDA and carbonyl levels by UV-C radiation was observed during refrigerated storage of sea bass fillets^19^ and tilapia fillets^4^. Some previous studies also found that an O_2_ scavenger retarded the lipid oxidation of rainbow trout fillets^14^, fresh cobia^13^ and sardines^15^ stored under refrigeration; however, only limited information is available regarding the effect of O_2_ scavengers on protein oxidation of fish species.

Two milligrams of MDA/kg is considered the limit above which meat is unfit for human consumption^55^. AP, OSP, AUV1, OSUV1, AUV3 and OSUV3 exceeded this limit on days 9, 19, 6, 19, 5 and 17, respectively (Supplementary Table S2). In spite of the importance of protein oxidation to food quality, there are no regulatory limits on carbonyl levels in meat products. In our study, when the acceptable microbial limit of 7 log CFU/g was reached, the carbonyl levels were 6.47 ± 0.06, 5.12 ± 0.00, 10.68 ± 0.00, 5.61 ± 0.40, 13.12 ± 0.22 and 5.58 ± 0.40 nmol of carbonyl/mg of protein for AP, OSP, AUV1, OSUV1, AUV3 and OSUV3, respectively (Supplementary Table S2). These results indicate the effectiveness of the O_2_ scavenger in retarding oxidative processes, even when oxidation-inducing treatments were used.

### Instrumental color measurements

Lightness (L*), redness (a*) and yellowness (b*) increased with the increasing storage period in all treatments (p < 0.05; Table 4). Throughout the entire storage period, no difference (p < 0.05) was found for L* values between treatments. AUV3 showed the highest (p < 0.05) a* and b* values during the entire storage period, followed by AUV1, AP, and treatments containing an O_2_ absorber (OSP, OSUV1 and OSUV3), which did not differ from each other (p > 0.05; Table 4). As in the lipid and protein oxidation, UV-C radiation increased the a* and b* values in a dose-dependent manner. The results of instrumental color parameters in all days of storage can be observed in Supplementary Table S3.

**Table 4:** Instrumental color parameters of tilapia (*Oreochromis niloticus*) fillets non- and treated with oxygen scavenger packaging (OSP) and ultraviolet radiation (UV-C) stored at 4 ± 1 °C for 23 days.

Parameters	Treatments^€^	AUC^¥^		Linear regression coefficients			
		AUC^¥^_0–13_	AUC^¥^_15–23_	y-intercept	slope	p-value	r-squared
L*	AP	739.10 ± 27.37^a^	NA	54.36 ± 0.54	0.37 ± 0.08	<0.0001	0.366
	OSP	730.30 ± 20.98^a^	476.40 ± 15.34^a^	54.17 ± 0.53	0.30 ± 0.04	<0.0001	0.469
	AUV1	726.80 ± 38.90^a^	481.20 ± 21.89^a^	53.44 ± 0.72	0.37 ± 0.06	<0.0001	0.423
	OSUV1	723.50 ± 27.49^a^	477.40 ± 16.77^a^	53.40 ± 0.54	0.35 ± 0.04	<0.0001	0.534
	AUV3	719.80 ± 29.39^a^	481.00 ± 14.66^a^	53.01 ± 0.52	0.38 ± 0.04	<0.0001	0.592
	OSUV3	720.90 ± 25.06^a^	476.90 ± 14.51^a^	53.17 ± 0.50	0.35 ± 0.04	<0.0001	0.578
a*	AP	32.12 ± 1.80^c^	NA	1.27 ± 0.06	0.19 ± 0.01	<0.0001	0.916
	OSP	25.17 ± 1.11^d^	28.08 ± 1.48^c^	1.24 ± 0.04	0.12 ± 0.00	<0.0001	0.954
	AUV1	38.35 ± 1.52^b^	40.59 ± 1.10^b^	1.71 ± 0.07	0.18 ± 0.01	<0.0001	0.953
	OSUV1	24.96 ± 1.46^d^	28.33 ± 1.34^c^	1.21 ± 0.04	0.12 ± 0.00	<0.0001	0.955
	AUV3	44.27 ± 1.87^a^	46.77 ± 1.65^a^	2.01 ± 0.07	0.21 ± 0.01	<0.0001	0.956
	OSUV3	25.35 ± 1.22^d^	28.68 ± 0.64^c^	1.23 ± 0.03	0.12 ± 0.00	<0.0001	0.971
b*	AP	84.33 ± 5.62^c^	NA	4.27 ± 0.13	0.33 ± 0.02	<0.0001	0.896
	OSP	73.09 ± 4.04^d^	63.33 ± 3.91^c^	4.24 ± 0.09	0.20 ± 0.01	<0.0001	0.928
	AUV1	94.82 ± 4.35^b^	81.84 ± 2.25^b^	5.14 ± 0.14	0.28 ± 0.01	<0.0001	0.913
	OSUV1	73.42 ± 3.90^d^	63.75 ± 1.68^c^	4.27 ± 0.07	0.20 ± 0.00	<0.0001	0.960
	AUV3	104.50 ± 3.67^a^	92.56 ± 3.00^a^	5.57 ± 0.16	0.33 ± 0.01	<0.0001	0.915
	OSUV3	73.77 ± 2.72^d^	63.70 ± 1.99^c^	4.32 ± 0.07	0.19 ± 0.01	<0.0001	0.953

Results are expressed as means ± standard deviation (n = 2). ^a,b,c,d^Different superscripts in the same column indicate significant differences (p < 0.05) among treatments. ^¥^AUC – Area under curve; AUC0-13 – from day 0 to 13 among treatments AP, OSP, AUV1, OSUV1, AUV3, and OSUV3; AUC15-23 – from day 15 to 23 among treatments OSP, AUV1, OSUV1, AUV3, and OSUV3. NA – Not applicable. ^€^AP (air packaging); OSP (oxygen scavenger packaging); AUV1 (air packaging + UV-C at 0.102 J/cm^2^); OSUV1 (oxygen scavenger packaging + UV-C at 0.102 J/cm^2^); AUV3 (air packaging + UV-C at 0.301 J/cm^2^); and OSUV3 (oxygen scavenger packaging + UV-C at 0.301 J/cm^2^).

The increase in lightness has been reported previously in freshwater fish species stored under refrigeration^4,56^, and has been associated with changes in the reflectance of the meat surface due to protein denaturation, exposing hydrophobic groups^57^. On the other hand, the increase in the a* and b* values in refrigerated white fish species leads to darkening, which has been related to discoloration^58^. It occurs due to myoglobin autoxidation, where ferrous iron (Fe^2+^) is oxidized to ferric iron (Fe^3+^), resulting in the formation and accumulation of metmyoglobin (MetMb)^8^. MDA can also contribute to an increase in MetMb accumulation by inactivating the metmyoglobin-reducing system and/or by interacting with myoglobin molecules through covalent bonds, which alters their primary structure, making myoglobin more susceptible to redox reactions^8,9^. In this study, the increase in the a* and b* values agrees with and can be explained by our results for lipid and protein oxidation, including the differences found among the treatments. Similarly, Monteiro *et al*.^4^ and Park & Ha^58^ observed that UV-C radiation increased a* and b* values in tilapia fillets and fresh chicken breast, respectively, over the refrigerated period. Chounou *et al*.^43^ reported that an O_2_ absorber was effective in preventing discoloration in ground meat stored under refrigeration.

### Instrumental texture parameters

Hardness, chewiness, cohesiveness, springiness and resilience decreased (p < 0.05) during the refrigerated period in all treatments (Table 5). OSP, OSUV1 and OSUV3 showed the highest (p < 0.05) hardness and chewiness, followed by samples submitted to air packaging (AP) and UV-C radiation at both doses (AUV1 and AUV3) during the storage period (Table 5). Cohesiveness, springiness and resilience were not affected (p > 0.05) by the O_2_ absorber and/or UV-C radiation, regardless of the dose, during the refrigerated storage period. The results of instrumental texture parameters in all days of storage can be found as Supplementary Table S4.

**Table 5:** Instrumental texture parameters of tilapia (*Oreochromis niloticus*) fillets non- and treated with oxygen scavenger packaging (OSP) and ultraviolet radiation (UV-C) stored at 4 ± 1 °C for 23 days.

Parameter	Treatments^€^	AUC^¥^		Linear regression coefficients			
		AUC^¥^_0–13_	AUC^¥^_15–23_	y-intercept	slope	p-value	r-squared
Hardness (g)	AP	40113.00 ± 507.00^b^	NA	4248.24 ± 74.65	−177.70 ± 10.92	<0.0001	0.923
	OSP	47535.00 ± 591.80^a^	18273.00 ± 438.40^b^	4570.65 ± 53.12	−97.70 ± 4.63	<0.0001	0.906
	AUV1	35239.00 ± 326.30^c^	12302.00 ± 211.70^a^	3604.38 ± 71.26	−112.82 ± 5.82	<0.0001	0.906
	OSUV1	47462.00 ± 249.30^a^	17574.00 ± 288.50^b^	4078.27 ± 35.60	−102.03 ± 2.92	<0.0001	0.965
	AUV3	35751.00 ± 301.40^c^	12012.00 ± 162.40^a^	3651.05 ± 66.53	−118.42 ± 5.52	<0.0001	0.911
	OSUV3	46852.00 ± 505.20^a^	17344.00 ± 194.60^b^	4103.58 ± 51.52	−106.18 ± 4.20	<0.0001	0.930
Chewiness (g × mm)	AP	8987.00 ± 104.40^b^	NA	1077.30 ± 55.60	−56.51 ± 8.52	<0.0001	0.647
	OSP	9724.00 ± 126.90^a^	3560.00 ± 86.95^b^	1149.48 ± 40.09	−33.96 ± 3.16	<0.0001	0.724
	AUV1	8166.00 ± 65.45^c^	2932.00 ± 25.87^a^	874.83 ± 40.14	−28.63 ± 3.25	<0.0001	0.648
	OSUV1	9676.00 ± 76.26^a^	3540.00 ± 15.95^b^	994.77 ± 39.66	−32.39 ± 3.45	<0.0001	0.710
	AUV3	8214.00 ± 65.28^c^	2906.00 ± 39.60^a^	889.79 ± 43.33	−29.62 ± 3.47	<0.0001	0.629
	OSUV3	9686.00 ± 77.45^a^	3501.00 ± 26.47^b^	1021.49 ± 39.39	−33.18 ± 3.11	<0.0001	0.736
Cohesiveness (ratio)	AP	5.22 ± 0.09^a^	NA	0.459 ± 0.007	−0.009 ± 0.001	<0.0001	0.627
	OSP	5.09 ± 0.09^a^	2.50 ± 0.04^a^	0.445 ± 0.005	−0.007 ± 0.000	<0.0001	0.845
	AUV1	5.20 ± 0.08^a^	2.57 ± 0.06^a^	0.449 ± 0.005	−0.007 ± 0.000	<0.0001	0.821
	OSUV1	5.33 ± 0.07^a^	2.61 ± 0.06^a^	0.459 ± 0.005	−0.007 ± 0.000	<0.0001	0.855
	AUV3	5.35 ± 0.10^a^	2.60 ± 0.07^a^	0.463 ± 0.006	−0.007 ± 0.000	<0.0001	0.839
	OSUV3	5.37 ± 0.09^a^	2.55 ± 0.05^a^	0.464 ± 0.005	−0.008 ± 0.000	<0.0001	0.863
Springiness (ratio)	AP	7.01 ± 0.13^a^	NA	0.596 ± 0.011	−0.008 ± 0.002	<0.0001	0.440
	OSP	7.10 ± 0.12^a^	3.71 ± 0.08^a^	0.599 ± 0.008	−0.007 ± 0.001	<0.0001	0.701
	AUV1	7.03 ± 0.07^a^	3.64 ± 0.08^a^	0.593 ± 0.007	−0.007 ± 0.001	<0.0001	0.766
	OSUV1	7.10 ± 0.13^a^	3.71 ± 0.09^a^	0.596 ± 0.009	−0.007 ± 0.001	<0.0001	0.662
	AUV3	7.14 ± 0.10^a^	3.71 ± 0.04^a^	0.599 ± 0.007	−0.007 ± 0.001	<0.0001	0.774
	OSUV3	7.16 ± 0.10^a^	3.66 ± 0.05^a^	0.601 ± 0.007	−0.007 ± 0.001	<0.0001	0.780
Resilience (ratio)	AP	1.80 ± 0.03^a^	NA	0.169 ± 0.004	−0.005 ± 0.001	<0.0001	0.692
	OSP	1.80 ± 0.03^a^	0.77 ± 0.02^a^	0.164 ± 0.003	−0.004 ± 0.000	<0.0001	0.854
	AUV1	1.82 ± 0.02^a^	0.77 ± 0.02^a^	0.167 ± 0.002	−0.004 ± 0.000	<0.0001	0.889
	OSUV1	1.83 ± 0.03^a^	0.81 ± 0.01^a^	0.168 ± 0.003	−0.004 ± 0.000	<0.0001	0.864
	AUV3	1.83 ± 0.03^a^	0.83 ± 0.02^a^	0.166 ± 0.002	−0.004 ± 0.000	<0.0001	0.853
	OSUV3	1.82 ± 0.02^a^	0.82 ± 0.02^a^	0.165 ± 0.002	−0.003 ± 0.000	<0.0001	0.859

Results are expressed as means ± standard deviation (n = 2). ^a,b,c,d^Different superscripts in the same column indicate significant differences (p < 0.05) among treatments. ^¥^AUC – Area under curve; AUC_0-13_ – from day 0 to 13 among treatments AP, OSP, AUV1, OSUV1, AUV3, and OSUV3; AUC_15–23_ – from day 15 to 23 among treatments OSP, AUV1, OSUV1, AUV3, and OSUV3. NA – Not applicable. ^€^AP (air packaging); OSP (oxygen scavenger packaging); AUV1 (air packaging + UV-C at 0.102 J/cm^2^); OSUV1 (oxygen scavenger packaging + UV-C at 0.102 J/cm^2^); AUV3 (air packaging + UV-C at 0.301 J/cm^2^); and OSUV3 (oxygen scavenger packaging + UV-C at 0.301 J/cm^2^).

Softening during the post-mortem period is related to the activity of endogenous and microbial proteolytic enzymes, which results in protein breakdown^59^. The results for hardness and chewiness in this study can be explained by the results for free amino acids, MDA level, carbonyl content, and TAPC. The pro-oxidant effect of the UV-C radiation increased the amount of free amino acids, indicating a higher proteolysis rate, while ROS formation at 0.102 and 0.301 J/cm^2^ was mitigated by O_2_ absorber. Furthermore, when compared to OSP, OSUV1 and OSUV3, both UV-C doses were less effective against growth of aerobic psychrotrophic bacteria, where *Pseudomonas* spp. is the dominant proteolytic spoilage bacteria in freshwater fish species^34^. There are no studies related to instrumental texture parameters in fish species packed with an O_2_ scavenger. However, in agreement with our study, Monteiro *et al*.^7^ reported that a similar UV-C dose decreased the hardness and chewiness but did not affect the cohesiveness and springiness of tilapia fillets stored under refrigeration. Molina *et al*.^19^ also observed that UV‐C treatment increased collagen degradation in sea bass fillets.

## Conclusion

The O_2_ scavenger, both UV-C doses (0.102 and 0.301 J/cm^2^) and combinations of these preservation methods, independently of the radiation dose, retarded the bacterial growth and the formation of TVB-N and ammonia, increasing the shelf life of refrigerated tilapia fillets by more than 50%, 60% and 70%, respectively. While UV-C doses induced adverse changes in the color, texture and oxidative processes, O_2_ scavenger demonstrated to be an effective and simple alternative to reduce the negative effects of UV-C radiation. Therefore, the O_2_ scavenger combined with UV-C radiation, regardless of the dose (0.102 or 0.301 J/cm^2^), was the most effective method to extend the shelf life and retard the loss of physicochemical quality of tilapia fillets stored under refrigeration.

## Supplementary information

Supplementary information.

Supplementary information2.

Supplementary information3.

Supplementary information4.
