# Supplementary material for: Combined effect of oxygen-scavenger packaging and UV-C radiation on shelf life of refrigerated tilapia (Oreochromis niloticus) fillets
Source: Sci Rep. 2020 Mar 6;10:4243. doi: 10.1038/s41598-020-61293-8 (PMC7060221; doi:10.1038/s41598-020-61293-8)
Supplement: Supplementary file 1 — Supplementary information. [file 41598_2020_61293_MOESM1_ESM.pdf]

Combined effect of oxygen-scavenger packaging and UV-C radiation on shelf life of refrigerated tilapia (*Oreochromis niloticus*) fillets

Maria Lúcia Guerra Monteiro, Eliane Teixeira Mársico, Yhan da Silva Mutz, Vinicius Silva Castro, Rodrigo Vilela de Barros Pinto Moreira, Thiago da Silveira Álvares & Carlos Adam Conte-Junior

Supplementary Table S2. Individual results of physicochemical parameters of tilapia (*Oreochromis niloticus*) fillets non- and treated with oxygen scavenger and ultraviolet radiation (UV-C) stored at 4 ± 1 °C for 23 days.

Days of storage	Ammonia (µg NH_3/g fish tissue)					
	Treatments€					
	AP	OSP	AUV1	OSUV1	AUV3	OSUV3
0	7.66±0.04	7.65±0.02	7.66±0.08	7.67±0.04	7.64±0.08	7.64±0.04
1	7.65±0.02	7.63±0.10	7.67±0.04	7.65±0.04	7.63±0.08	7.65±0.04
2	8.32±0.01	7.89±0.01	8.19±0.06	7.69±0.01	8.15±0.04	7.71±0.03
3	8.43±0.10	8.03±0.01	8.22±0.09	7.79±0.02	8.21±0.10	7.81±0.07
4	8.98±0.04	8.25±0.01	8.51±0.04	7.88±0.01	8.50±0.06	7.90±0.10
5	9.49±0.24	8.42±0.05	9.14±0.18	8.12±0.07	9.13±0.06	8.09±0.09
6	10.14±0.06	9.23±0.01	9.52±0.05	8.41±0.05	9.55±0.08	8.43±0.16
9	10.60±0.03	9.89±0.06	10.20±0.11	8.75±0.01	10.24±0.05	8.79±0.09
11	11.78±0.08	10.22±0.10	10.89±0.11	9.23±0.05	10.91±0.18	9.20±0.06
13	12.81±0.23	10.28±0.09	10.92±0.11	9.60±0.14	10.96±0.03	9.64±0.11
15	NA	10.79±0.18	11.46±0.05	9.89±0.08	11.47±0.03	9.82±0.12
17	NA	10.97±0.06	11.52±0.09	10.11±0.05	11.58±0.11	10.14±0.06
19	NA	11.33±0.05	11.87±0.03	10.57±0.03	11.85±0.09	10.58±0.04
21	NA	11.53±0.04	11.97±0.02	10.80±0.06	11.96±0.06	10.85±0.10
23	NA	11.89±0.03	12.28±0.13	11.19±0.08	12.25±0.08	11.22±0.05
Days of storage	Total volatile basic nitrogen (mg TVB-N/100g fish tissue)					

	Treatments€					
	AP	OSP	AUV1	OSUV1	AUV3	OSUV3
0	10.08±0.00	10.08±0.00	10.08±0.00	10.08±0.00	10.08±0.00	10.08±0.08
1	10.08±0.00	10.08±0.00	10.23±0.21	10.08±0.00	10.08±0.00	10.08±0.08
2	15.02±0.76	11.95±0.18	13.01±0.13	10.18±0.14	13.06±0.17	10.13±0.07
3	15.47±0.18	12.07±0.07	13.32±0.28	11.08±0.17	13.35±0.07	11.15±0.22
4	16.10±0.71	12.99±0.86	14.28±0.45	11.64±0.51	14.33±0.21	11.67±0.07
5	16.74±0.88	13.79±0.21	14.74±0.06	11.75±0.21	14.71±0.23	11.79±0.04
6	17.62±0.34	14.34±0.00	15.49±0.52	12.07±0.92	15.40±0.40	12.10±0.57
9	17.75±0.89	14.98±0.40	15.95±0.06	12.38±0.37	15.92±0.08	12.41±0.07
11	18.75±0.52	15.31±0.35	16.64±0.31	12.97±0.52	16.69±0.33	12.94±0.17
13	20.92±1.07	15.79±0.16	17.27±0.75	13.16±0.42	17.20±0.20	13.20±0.06
15	NA	15.77±0.04	17.35±0.81	13.67±0.95	17.39±0.10	13.74±0.40
17	NA	16.42±0.42	18.73±0.18	14.47±0.72	18.77±0.07	14.40±0.34
19	NA	20.56±0.57	22.73±0.13	15.89±0.04	22.79±0.38	15.86±0.14
21	NA	20.61±0.64	23.39±0.66	16.46±0.57	23.42±0.06	16.54±0.19
23	NA	21.62±0.65	23.71±0.47	18.29±0.49	23.76±0.06	18.34±0.31
	Lipid oxidation (mg malondialdehyde/kg fish tissue)					
Days of storage	Treatments€					
	AP	OSP	AUV1	OSUV1	AUV3	OSUV3
0	0.24±0.00	0.24±0.00	0.24±0.00	0.26±0.02	0.25±0.02	0.26±0.02
1	0.42±0.01	0.28±0.01	0.54±0.01	0.28±0.01	0.64±0.01	0.30±0.01
2	0.44±0.01	0.27±0.00	0.56±0.01	0.29±0.03	0.67±0.03	0.31±0.03
3	0.50±0.01	0.37±0.04	0.61±0.03	0.38±0.01	1.04±0.00	0.41±0.03
4	0.62±0.01	0.44±0.01	0.90±0.08	0.46±0.04	1.48±0.07	0.46±0.04
5	0.91±0.07	0.46±0.01	1.39±0.11	0.50±0.04	2.10±0.08	0.55±0.03
6	1.56±0.12	0.65±0.03	2.04±0.05	0.69±0.05	2.66±0.06	0.66±0.01
9	2.10±0.01	0.88±0.00	2.37±0.06	0.90±0.03	2.85±0.02	0.92±0.03
11	2.61±0.02	1.44±0.04	2.97±0.05	1.46±0.01	3.00±0.19	1.50±0.04
13	2.88±0.04	1.52±0.04	3.09±0.05	1.53±0.03	3.52±0.00	1.58±0.04

15	NA	1.87±0.07	3.28±0.04	1.93±0.01	3.53±0.08	1.91±0.04
17	NA	2.00±0.03	3.47±0.06	1.99±0.04	3.89±0.01	2.02±0.03
19	NA	2.72±0.03	3.63±0.01	2.73±0.04	4.05±0.01	2.74±0.06
21	NA	3.02±0.04	3.74±0.06	3.05±0.08	4.36±0.03	3.05±0.13
23	NA	3.56±0.06	3.94±0.01	3.58±0.14	4.57±0.03	3.54±0.21

Days of storage	Protein oxidation (nmol carbonyls/mg protein)					
	Treatments[€]					
	AP	OSP	AUV1	OSUV1	AUV3	OSUV3
0	1.22±0.08	1.31±0.09	1.28±0.08	1.22±0.08	1.30±0.08	1.33±0.10
1	2.47±0.16	1.36±0.03	3.53±0.28	1.38±0.11	4.41±0.21	1.41±0.11
2	3.04±0.11	1.94±0.08	4.69±0.13	2.12±0.16	4.98±0.04	2.06±0.16
3	2.84±0.14	2.01±0.12	5.90±0.22	2.03±0.14	6.52±0.22	2.14±0.18
4	4.34±0.33	3.14±0.12	6.03±0.27	3.19±0.14	6.76±0.29	3.26±0.17
5	4.93±0.27	3.51±0.08	6.70±0.14	3.52±0.18	7.34±0.27	3.67±0.18
6	5.82±0.03	3.83±0.06	6.86±0.05	3.92±0.12	7.37±0.11	3.94±0.15
9	6.47±0.06	3.93±0.08	7.48±0.08	3.90±0.06	8.60±0.21	4.00±0.25
11	7.81±0.12	4.30±0.17	8.95±0.13	4.26±0.24	9.65±0.20	4.23±0.20
13	9.44±0.15	5.06±0.08	10.27±0.06	5.11±0.06	11.93±0.30	5.18±0.12
15	NA	5.20±0.11	10.68±0.00	5.26±0.10	13.12±0.44	5.15±0.19
17	NA	5.86±0.12	11.83±0.03	5.94±0.07	14.34±0.85	5.96±0.13
19	NA	5.70±0.11	11.60±0.16	5.75±0.13	14.25±0.89	5.78±0.13
21	NA	6.48±0.11	13.13±0.67	6.56±0.08	15.31±0.73	6.44±0.21
23	NA	7.06±0.23	13.34±0.38	7.03±0.11	16.32±0.57	7.18±0.22

Results are expressed as means ± standard deviation (n = 2). NA – Not applicable. [€]AP (air packaging); OSP (oxygen scavenger packaging); AUV1 (air packaging + UV-C at 0.102 J/cm^2); OSUV1 (oxygen scavenger packaging + UV-C at 0.102 J/cm^2); AUV3 (air packaging + UV-C at 0.301 J/cm^2); and OSUV3 (oxygen scavenger packaging + UV-C at 0.301 J/cm^2).
