# Supplementary material for: Combined effect of oxygen-scavenger packaging and UV-C radiation on shelf life of refrigerated tilapia (Oreochromis niloticus) fillets
Source: Sci Rep. 2020 Mar 6;10:4243. doi: 10.1038/s41598-020-61293-8 (PMC7060221; doi:10.1038/s41598-020-61293-8)
Supplement: Supplementary file 2 — Supplementary information2. [file 41598_2020_61293_MOESM2_ESM.pdf]

Combined effect of oxygen-scavenger packaging and UV-C radiation on shelf life of refrigerated tilapia (*Oreochromis niloticus*) fillets

Maria Lúcia Guerra Monteiro, Eliane Teixeira Mársico, Yhan da Silva Mutz, Vinicius Silva Castro, Rodrigo Vilela de Barros Pinto Moreira, Thiago da Silveira Álvares & Carlos Adam Conte-Junior

Supplementary Table S3. Individual results of instrumental color parameters of tilapia (*Oreochromis niloticus*) fillets non- and treated with oxygen scavenger and ultraviolet radiation (UV-C) stored at 4 ± 1 °C for 23 days.

Days of storage	Lightness (L^*)					
	Treatments[€]					
	AP	OSP	AUV1	OSUV1	AUV3	OSUV3
0	52.04±0.88	53.75±3.35	52.14±4.76	52.63±2.71	54.09±2.40	54.67±0.99
1	55.18±1.09	54.20±3.74	54.91±2.29	54.06±3.89	54.23±2.43	53.47±1.99
2	55.96±1.70	54.18±2.97	54.19±2.17	54.84±2.26	54.00±2.21	54.26±3.55
3	56.30±1.57	55.53±3.20	54.13±3.85	54.30±3.38	55.58±3.02	54.54±0.69
4	55.88±1.71	56.17±1.90	55.77±3.94	55.02±1.47	54.53±1.58	55.57±2.39
5	56.90±2.12	56.92±3.28	55.79±3.28	53.96±3.25	54.61±1.80	54.36±1.36
6	57.11±3.32	56.74±0.49	56.90±4.99	54.25±3.88	54.20±2.09	55.51±1.28
9	57.57±2.11	56.75±1.31	56.51±2.97	56.51±2.15	55.05±1.24	56.36±3.02
11	57.88±3.41	56.94±2.21	56.65±3.42	56.69±1.77	56.64±4.39	56.76±1.25
13	58.99±1.55	57.20±1.31	57.56±3.00	57.89±0.42	56.35±1.47	58.27±5.09
15	NA	58.92±2.56	58.11±1.27	58.84±3.66	58.94±3.90	56.58±0.55
17	NA	58.36±2.64	59.96±5.03	58.24±4.05	58.10±1.98	58.33±4.64
19	NA	59.05±3.27	60.03±2.98	59.64±2.50	60.28±1.65	58.16±0.97
21	NA	59.70±0.57	59.37±3.45	61.25±1.27	60.63±0.58	60.75±0.91
23	NA	63.24±2.13	64.40±1.45	63.51±2.17	65.95±1.01	62.84±0.89
Days of storage	Redness (a^*)					

	Treatments€					
	AP	OSP	AUV1	OSUV1	AUV3	OSUV3
0	1.48±0.14	1.46±0.14	1.49±0.07	1.46±0.06	1.49±0.09	1.47±0.10
1	1.56±0.06	1.45±0.11	1.81±0.06	1.47±0.11	2.17±0.20	1.49±0.05
2	1.67±0.07	1.51±0.11	1.89±0.05	1.53±0.13	2.15±0.10	1.56±0.07
3	1.83±0.09	1.50±0.12	2.20±0.12	1.52±0.13	2.72±0.08	1.58±0.12
4	1.94±0.12	1.69±0.10	2.32±0.08	1.65±0.14	2.78±0.18	1.68±0.09
5	2.01±0.05	1.81±0.07	2.67±0.09	1.82±0.09	3.11±0.10	1.83±0.08
6	2.24±0.17	1.89±0.05	2.82±0.24	1.81±0.12	3.54±0.24	1.84±0.11
9	2.73±0.22	2.18±0.09	3.51±0.16	2.14±0.18	4.00±0.21	2.18±0.13
11	3.41±0.17	2.37±0.18	3.72±0.05	2.36±0.08	4.11±0.04	2.39±0.13
13	4.26±0.35	2.53±0.13	4.86±0.16	2.55±0.17	5.33±0.11	2.59±0.07
15	NA	2.84±0.08	4.80±0.16	2.90±0.17	5.47±0.22	2.89±0.11
17	NA	3.27±0.21	4.89±0.10	3.33±0.14	5.46±0.18	3.37±0.05
19	NA	3.40±0.16	5.08±0.16	3.51±0.25	5.90±0.11	3.58±0.13
21	NA	3.96±0.30	5.18±0.34	3.85±0.17	6.10±0.45	3.90±0.10
23	NA	4.00±0.35	5.51±0.09	4.05±0.33	6.38±0.09	4.10±0.10
	Yellowness (*b**)					
Days of storage	Treatments€					
	AP	OSP	AUV1	OSUV1	AUV3	OSUV3
0	4.02±0.32	3.97±0.21	4.09±0.26	3.99±0.24	4.06±0.40	4.02±0.34
1	4.44±0.28	4.21±0.12	4.78±0.18	4.24±0.12	5.28±0.47	4.32±0.26
2	4.71±0.11	4.50±0.34	5.18±0.20	4.58±0.19	5.77±0.12	4.52±0.22
3	5.36±0.14	4.96±0.12	6.04±0.31	4.91±0.41	6.50±0.26	4.98±0.39
4	5.58±0.25	5.02±0.15	6.36±0.35	5.10±0.18	7.22±0.20	5.19±0.18
5	6.40±0.60	5.40±0.25	7.24±0.26	5.45±0.11	8.24±0.46	5.49±0.08
6	6.58±0.30	5.78±0.26	7.35±0.25	5.83±0.21	8.36±0.62	5.85±0.32
9	7.39±0.61	6.23±0.54	8.34±0.61	6.19±0.61	9.21±0.11	6.26±0.15
11	8.03±0.73	6.63±0.40	9.16±0.34	6.66±0.08	9.87±0.09	6.60±0.27
13	8.17±0.61	6.65±0.28	9.32±0.33	6.69±0.18	10.01±0.16	6.72±0.28

15	NA	7.02±0.30	9.53±0.12	7.09±0.11	10.61±0.16	7.15±0.11
17	NA	7.68±0.70	10.02±0.09	7.74±0.26	11.23±0.51	7.80±0.06
19	NA	7.86±0.77	10.11±0.68	7.79±0.20	11.34±0.69	7.63±0.35
21	NA	8.22±0.63	10.58±0.19	8.36±0.33	12.26±0.33	8.46±0.51
23	NA	8.81±0.08	10.91±0.07	8.91±0.33	12.30±0.31	8.79±0.12

Results are expressed as means ± standard deviation (n = 2). NA – Not applicable. [€]AP (air packaging); OSP (oxygen scavenger packaging); AUV1 (air packaging + UV-C at 0.102 J/cm^2); OSUV1 (oxygen scavenger packaging + UV-C at 0.102 J/cm^2); AUV3 (air packaging + UV-C at 0.301 J/cm^2); and OSUV3 (oxygen scavenger packaging + UV-C at 0.301 J/cm^2).
