# Supplementary material for: Combined effect of oxygen-scavenger packaging and UV-C radiation on shelf life of refrigerated tilapia (Oreochromis niloticus) fillets
Source: Sci Rep. 2020 Mar 6;10:4243. doi: 10.1038/s41598-020-61293-8 (PMC7060221; doi:10.1038/s41598-020-61293-8)
Supplement: Supplementary file 3 — Supplementary information3. [file 41598_2020_61293_MOESM3_ESM.pdf]

Combined effect of oxygen-scavenger packaging and UV-C radiation on shelf life of refrigerated tilapia (*Oreochromis niloticus*) fillets

Maria Lúcia Guerra Monteiro, Eliane Teixeira Mársico, Yhan da Silva Mutz, Vinicius Silva Castro, Rodrigo Vilela de Barros Pinto Moreira, Thiago da Silveira Álvares & Carlos Adam Conte-Junior

Supplementary Table S4. Individual results of instrumental texture parameters of tilapia (*Oreochromis niloticus*) fillets non- and treated with oxygen scavenger and ultraviolet radiation (UV-C) stored at 4 ± 1 °C for 23 days.

Days of storage	Hardness (g)					
	Treatments[€]					
	AP	OSP	AUV1	OSUV1	AUV3	OSUV3
0	4406.66±388.29	4436.71±232.88	4302.81±355.63	4411.41±60.57	4311.85±214.36	4480.95±281.40
1	4004.51±269.29	4540.46±262.45	3520.02±104.57	3922.35±26.78	3568.20±79.41	3920.73±56.43
2	4045.57±197.48	4485.45±245.45	3452.34±78.98	3916.12±86.46	3578.67±85.71	3893.93±45.50
3	3553.39±209.21	4563.17±383.05	3122.38±114.58	3862.08±76.23	3268.68±78.70	3875.43±38.65
4	3390.34±134.94	4105.25±223.89	2926.01±54.41	3716.69±122.62	2888.43±22.55	3783.37±68.87
5	3315.49±181.95	3979.05±183.95	2935.22±68.47	3521.93±39.24	2969.40±88.38	3501.86±63.72
6	3195.63±86.57	3898.99±198.07	2795.63±153.92	3364.40±121.22	2769.63±77.81	3261.85±153.95
9	2515.91±175.35	3507.56±209.31	2135.83±95.70	2872.55±49.88	2208.32±64.87	2722.29±231.35
11	2413.47±175.49	3401.54±45.41	2161.66±74.33	2876.78±86.84	2246.92±182.74	2809.83±94.62
13	2092.58±119.95	3273.41±87.96	1877.54±55.25	2652.98±106.42	1767.39±107.89	2687.57±77.46
15	NA	3078.12±61.32	1765.93±55.06	2597.51±52.36	1737.47±140.28	2613.15±134.82
17	NA	2896.42±258.81	1644.11±58.72	2330.47±105.78	1598.45±29.08	2297.14±59.80
19	NA	2725.28±204.93	1593.07±115.10	2291.03±174.63	1496.23±82.66	2277.71±59.99
21	NA	2660.77±251.46	1381.19±98.40	1977.47±95.17	1385.36±37.58	1936.93±46.36
23	NA	2368.27±110.17	1299.40±107.08	1778.64±96.93	1314.11±16.63	1707.04±85.52
Days of storage	Chewiness (g × mm)					

Days of storage	AP	OSP	AUV1	OSUV1	AUV3	OSUV3
	Treatments€					
	AP	OSP	AUV1	OSUV1	AUV3	OSUV3
0	1451.43±74.16	1419.53±92.45	1406.08±77.07	1421.95±103.85	1473.88±68.03	1403.00±93.34
1	946.07±2.93	1443.91±95.79	817.60±5.96	944.09±36.72	825.62±18.82	971.01±17.51
2	917.04±22.23	1137.61±39.12	736.60±17.47	928.85±30.38	720.63±27.15	929.87±54.96
3	797.48±34.69	934.82±45.66	725.18±22.58	823.30±26.64	719.38±2.04	827.98±40.74
4	743.95±54.03	907.95±55.84	710.53±16.66	807.06±12.91	711.35±8.57	802.79±16.63
5	636.21±50.35	788.72±34.86	583.65±33.58	679.11±25.55	587.92±21.65	670.07±48.96
6	590.18±56.93	753.81±15.96	532.06±18.76	650.21±31.37	543.70±15.06	634.60±39.17
9	582.10±21.37	750.00±32.42	521.33±3.52	603.69±4.71	526.41±12.82	608.50±6.65
11	555.15±11.77	642.93±20.05	535.98±14.02	590.51±11.63	531.62±26.06	592.81±19.88
13	474.87±29.60	642.16±28.14	441.57±22.18	502.75±8.33	447.96±9.80	508.42±11.50
15	NA	625.12±41.52	434.08±4.30	504.62±12.62	435.44±16.00	507.76±10.46
17	NA	641.41±20.95	397.95±8.58	497.56±3.54	396.99±6.27	487.02±10.58
19	NA	591.25±58.00	370.38±1.85	436.94±8.26	366.50±21.21	421.81±3.05
21	NA	477.19±17.56	331.58±10.89	404.61±7.08	324.45±9.47	407.03±11.03
23	NA	459.60±17.12	296.65±12.54	356.96±9.40	293.66±10.38	361.33±10.29
	Cohesiveness (ratio)					
Days of storage	Treatments€					
	AP	OSP	AUV1	OSUV1	AUV3	OSUV3
0	0.458±0.025	0.454±0.033	0.464±0.026	0.463±0.015	0.463±0.018	0.467±0.023
1	0.449±0.021	0.444±0.025	0.453±0.041	0.462±0.032	0.457±0.044	0.455±0.018
2	0.441±0.038	0.440±0.018	0.448±0.022	0.443±0.014	0.459±0.014	0.458±0.024
3	0.440±0.042	0.433±0.034	0.421±0.016	0.436±0.017	0.450±0.023	0.451±0.031
4	0.422±0.033	0.422±0.025	0.413±0.024	0.424±0.009	0.427±0.024	0.420±0.027
5	0.420±0.040	0.400±0.017	0.413±0.004	0.436±0.038	0.433±0.023	0.436±0.021
6	0.411±0.037	0.385±0.026	0.385±0.028	0.397±0.020	0.403±0.039	0.406±0.004
9	0.372±0.027	0.352±0.024	0.371±0.021	0.391±0.016	0.389±0.034	0.386±0.035
11	0.360±0.019	0.358±0.036	0.379±0.034	0.380±0.026	0.374±0.013	0.385±0.033
13	0.352±0.023	0.359±0.023	0.365±0.015	0.363±0.014	0.365±0.028	0.373±0.034

15	NA	0.341±0.006	0.331±0.025	0.342±0.034	0.346±0.030	0.337±0.012
17	NA	0.329±0.023	0.345±0.030	0.353±0.019	0.347±0.032	0.341±0.021
19	NA	0.304±0.017	0.315±0.011	0.319±0.018	0.318±0.011	0.309±0.017
21	NA	0.304±0.009	0.312±0.024	0.321±0.025	0.314±0.024	0.316±0.016
23	NA	0.282±0.011	0.291±0.006	0.282±0.017	0.297±0.024	0.284±0.011
	Springiness (ratio)					
Days of storage	Treatments€					
	AP	OSP	AUV1	OSUV1	AUV3	OSUV3
0	0.649±0.020	0.656±0.045	0.639±0.027	0.642±0.024	0.647±0.046	0.649±0.030
1	0.575±0.047	0.598±0.044	0.576±0.042	0.582±0.040	0.584±0.019	0.589±0.013
2	0.560±0.046	0.566±0.019	0.570±0.038	0.576±0.047	0.572±0.010	0.580±0.021
3	0.559±0.034	0.563±0.029	0.563±0.026	0.565±0.054	0.565±0.035	0.566±0.037
4	0.552±0.051	0.542±0.034	0.556±0.031	0.550±0.050	0.553±0.025	0.560±0.032
5	0.544±0.032	0.558±0.049	0.549±0.029	0.541±0.012	0.543±0.012	0.546±0.030
6	0.539±0.031	0.540±0.043	0.534±0.013	0.545±0.050	0.544±0.014	0.536±0.043
9	0.526±0.036	0.534±0.050	0.527±0.003	0.526±0.027	0.533±0.022	0.533±0.032
11	0.501±0.049	0.508±0.031	0.503±0.022	0.519±0.049	0.528±0.049	0.530±0.019
13	0.501±0.036	0.513±0.016	0.508±0.032	0.522±0.040	0.529±0.031	0.529±0.022
15	NA	0.496±0.028	0.490±0.039	0.500±0.050	0.499±0.014	0.492±0.038
17	NA	0.461±0.026	0.456±0.022	0.472±0.025	0.474±0.013	0.468±0.020
19	NA	0.467±0.032	0.446±0.044	0.447±0.014	0.451±0.023	0.445±0.009
21	NA	0.463±0.025	0.457±0.016	0.466±0.039	0.466±0.012	0.461±0.017
23	NA	0.430±0.034	0.427±0.026	0.434±0.024	0.426±0.013	0.422±0.017
	Resilience (ratio)					
Days of storage	Treatments€					
	AP	OSP	AUV1	OSUV1	AUV3	OSUV3
0	0.183±0.017	0.182±0.015	0.182±0.015	0.183±0.015	0.178±0.012	0.185±0.012
1	0.174±0.014	0.171±0.014	0.167±0.014	0.165±0.012	0.165±0.007	0.162±0.016
2	0.156±0.013	0.152±0.013	0.159±0.014	0.160±0.014	0.160±0.010	0.158±0.008
3	0.146±0.010	0.149±0.006	0.153±0.010	0.154±0.012	0.156±0.014	0.153±0.007

4	0.141±0.013	0.138±0.011	0.140±0.008	0.143±0.010	0.142±0.010	0.140±0.003
5	0.135±0.011	0.136±0.009	0.139±0.007	0.139±0.010	0.144±0.011	0.144±0.012
6	0.136±0.006	0.133±0.010	0.137±0.005	0.140±0.014	0.136±0.008	0.138±0.010
9	0.132±0.010	0.135±0.008	0.131±0.007	0.134±0.010	0.131±0.012	0.130±0.003
11	0.122±0.008	0.120±0.004	0.124±0.004	0.125±0.010	0.127±0.008	0.125±0.006
13	0.114±0.008	0.117±0.011	0.116±0.009	0.113±0.006	0.116±0.010	0.116±0.009
15	NA	0.109±0.010	0.110±0.005	0.116±0.006	0.115±0.008	0.113±0.005
17	NA	0.105±0.008	0.103±0.009	0.110±0.002	0.116±0.011	0.111±0.012
19	NA	0.100±0.005	0.103±0.010	0.107±0.007	0.109±0.008	0.105±0.005
21	NA	0.087±0.005	0.088±0.007	0.092±0.003	0.092±0.006	0.095±0.005
23	NA	0.073±0.006	0.075±0.005	0.075±0.005	0.078±0.007	0.079±0.007

Results are expressed as means ± standard deviation (n = 2). NA – Not applicable. [€]AP (air packaging); OSP (oxygen scavenger packaging); AUV1 (air packaging + UV-C at 0.102 J/cm^2); OSUV1 (oxygen scavenger packaging + UV-C at 0.102 J/cm^2); AUV3 (air packaging + UV-C at 0.301 J/cm^2); and OSUV3 (oxygen scavenger packaging + UV-C at 0.301 J/cm^2).
