# Supplementary material for: Combined effect of oxygen-scavenger packaging and UV-C radiation on shelf life of refrigerated tilapia (Oreochromis niloticus) fillets
Source: Sci Rep. 2020 Mar 6;10:4243. doi: 10.1038/s41598-020-61293-8 (PMC7060221; doi:10.1038/s41598-020-61293-8)
Supplement: Supplementary file 4 — Supplementary information4. [file 41598_2020_61293_MOESM4_ESM.pdf]

Combined effect of oxygen-scavenger packaging and UV-C radiation on shelf life of refrigerated tilapia (*Oreochromis niloticus*) fillets

Maria Lúcia Guerra Monteiro, Eliane Teixeira Mársico, Yhan da Silva Mutz, Vinicius Silva Castro, Rodrigo Vilela de Barros Pinto Moreira, Thiago da Silveira Álvares & Carlos Adam Conte-Junior

Supplementary Table S1. Individual results of free amino acids and biogenic amines of tilapia (*Oreochromis niloticus*) fillets non- and treated with oxygen scavenger and ultraviolet radiation (UV-C) stored at 4 ± 1 °C for 23 days.

Days of storage	L-lysine (mg lysine/kg fish tissue)					
	Treatments€					
	AP	OSP	AUV1	OSUV1	AUV3	OSUV3
0	8.42±0.25	8.46±0.31	10.94±0.25	10.86±0.74	10.97±0.35	10.80±0.44
1	12.73±0.15	10.88±0.88	14.37±0.75	10.89±0.08	14.23±0.15	10.76±0.21
2	16.34±0.77	13.19±0.75	18.58±0.37	13.01±0.93	17.83±0.70	13.12±0.13
3	20.27±0.95	15.64±0.62	23.39±0.54	15.71±0.32	23.82±0.29	15.75±0.55
4	24.47±0.25	17.97±0.97	26.85±0.75	17.92±0.15	26.50±0.65	17.98±0.40
5	26.73±0.34	21.06±0.87	29.54±0.61	21.09±0.08	29.22±0.43	21.13±0.30
6	29.23±0.37	24.99±1.26	34.04±0.58	24.83±0.24	34.06±0.12	24.93±0.30
9	34.03±0.43	28.29±0.16	36.89±0.76	28.36±0.47	36.64±0.36	28.47±0.66
11	38.51±0.44	32.17±0.78	40.06±0.98	32.29±0.22	40.17±0.12	32.28±0.19
13	41.97±0.12	34.39±0.35	44.28±0.78	34.49±0.26	44.13±0.24	34.50±0.40
15	NA	36.32±0.27	46.00±0.06	36.26±0.15	46.15±0.38	36.32±0.38
17	NA	37.86±0.33	47.14±0.63	37.77±0.38	47.18±0.13	37.84±0.04
19	NA	40.09±0.52	49.25±0.37	40.11±0.36	49.36±0.09	40.04±0.17
21	NA	43.05±0.74	52.00±0.74	43.01±0.14	52.29±0.12	43.08±0.13
23	NA	45.85±0.59	53.45±0.68	45.81±0.04	53.81±0.38	45.96±0.30
Days of storage	L-ornithine (mg ornitine/kg fish tissue)					

	Treatments€					
	AP	OSP	AUV1	OSUV1	AUV3	OSUV3
0	1.39±0.12	1.40±0.03	1.95±0.07	1.83±0.07	1.94±0.08	1.85±0.10
1	1.82±0.03	1.58±0.04	2.11±0.05	1.78±0.05	2.13±0.07	1.83±0.10
2	2.18±0.10	1.71±0.04	2.62±0.12	1.82±0.11	2.65±0.08	1.76±0.08
3	2.99±0.07	1.96±0.06	3.29±0.06	2.01±0.01	3.33±0.08	2.01±0.06
4	3.26±0.22	2.31±0.12	3.73±0.12	2.33±0.11	3.66±0.13	2.36±0.13
5	3.64±0.08	2.60±0.03	3.98±0.03	2.63±0.18	3.95±0.06	2.68±0.12
6	3.84±0.10	2.86±0.17	4.21±0.11	2.84±0.19	4.20±0.09	2.86±0.07
9	4.28±0.01	3.01±0.06	4.61±0.02	3.05±0.07	4.63±0.15	3.06±0.02
11	4.63±0.04	3.20±0.04	4.92±0.10	3.24±0.17	4.90±0.12	3.25±0.02
13	4.83±0.10	3.63±0.04	5.16±0.03	3.57±0.09	5.12±0.10	3.61±0.10
15	NA	3.81±0.12	5.53±0.11	3.87±0.12	5.59±0.09	3.84±0.16
17	NA	4.06±0.11	5.84±0.02	4.08±0.05	5.87±0.14	4.10±0.07
19	NA	4.17±0.07	5.96±0.06	4.22±0.11	5.99±0.01	4.25±0.12
21	NA	4.33±0.13	6.18±0.11	4.32±0.20	6.17±0.15	4.29±0.17
23	NA	4.61±0.07	6.42±0.15	4.64±0.16	6.45±0.15	4.69±0.09
Days of storage	L-arginine (mg arginine/kg fish tissue)					
	Treatments€					
	AP	OSP	AUV1	OSUV1	AUV3	OSUV3
0	9.13±0.03	9.06±0.19	9.43±0.04	9.42±0.14	9.42±0.14	9.50±0.12
1	9.34±0.14	9.20±0.11	9.76±0.05	9.39±0.12	9.80±0.11	9.38±0.30
2	13.14±0.62	10.34±0.07	17.76±1.57	10.40±0.24	17.65±0.81	10.36±0.34
3	16.13±0.50	12.68±0.91	19.92±0.74	12.23±0.28	20.07±0.14	12.35±0.09
4	22.32±0.89	14.65±0.65	24.59±0.55	14.71±0.72	24.69±0.90	14.77±0.20
5	25.51±0.40	17.39±1.56	27.77±0.63	17.18±0.56	27.88±0.60	17.67±0.22
6	29.01±0.16	20.73±1.23	33.37±0.84	20.67±0.60	33.75±1.00	20.48±0.26
9	32.60±1.21	21.94±0.98	35.49±0.06	21.96±0.57	35.46±0.22	21.91±0.56
11	34.96±3.08	25.76±0.16	36.74±0.78	25.86±0.32	36.60±0.12	25.78±0.18
13	37.33±0.06	27.52±1.55	40.20±0.19	27.63±0.29	40.28±0.92	27.67±0.33

15	NA	29.33±1.62	44.19±0.59	29.38±0.24	44.08±0.60	29.43±0.37
17	NA	32.99±2.70	46.93±1.30	32.85±0.17	46.76±1.41	32.73±0.20
19	NA	34.20±0.37	49.34±0.75	34.36±0.40	49.16±0.63	34.47±0.20
21	NA	36.79±0.80	51.95±1.06	36.36±0.53	51.90±0.25	36.20±0.70
23	NA	37.74±1.23	52.94±1.02	37.73±0.23	52.64±0.84	37.75±0.22

Days of storage	Cadaverine (mg cadaverine/kg fish tissue)					
	Treatments[€]					
	AP	OSP	AUV1	OSUV1	AUV3	OSUV3
0	1.89±0.09	1.90±0.03	1.86±0.09	1.88±0.05	1.91±0.05	1.88±0.08
1	2.23±0.07	1.96±0.06	2.24±0.09	1.95±0.04	2.27±0.06	1.91±0.09
2	2.50±0.18	2.07±0.11	2.56±0.04	2.08±0.08	2.59±0.12	2.10±0.08
3	2.82±0.14	2.33±0.09	2.85±0.08	2.37±0.11	2.87±0.05	2.33±0.10
4	3.12±0.17	2.50±0.05	3.16±0.12	2.45±0.09	3.15±0.16	2.46±0.08
5	3.55±0.04	2.70±0.07	3.51±0.14	2.72±0.10	3.56±0.06	2.76±0.10
6	3.83±0.02	2.78±0.02	3.82±0.11	2.81±0.08	3.81±0.13	2.83±0.09
9	4.16±0.17	3.12±0.09	4.20±0.09	3.14±0.12	4.22±0.09	3.17±0.10
11	4.47±0.18	3.57±0.11	4.48±0.07	3.55±0.08	4.49±0.04	3.58±0.04
13	4.82±0.29	3.81±0.09	4.87±0.04	3.87±0.05	4.80±0.09	3.89±0.05
15	NA	3.97±0.04	5.05±0.01	4.03±0.07	5.06±0.16	4.01±0.06
17	NA	4.09±0.07	5.31±0.04	4.05±0.05	5.32±0.04	4.07±0.04
19	NA	4.25±0.01	5.35±0.03	4.23±0.13	5.38±0.08	4.28±0.10
21	NA	4.57±0.10	5.84±0.11	4.52±0.11	5.81±0.10	4.50±0.14
23	NA	4.60±0.07	5.97±0.04	4.65±0.08	5.93±0.05	4.68±0.08

Days of storage	Putrescine (mg putrescine/kg fish tissue)					
	Treatments[€]					
	AP	OSP	AUV1	OSUV1	AUV3	OSUV3
0	12.00±0.04	11.75±0.59	12.05±0.11	12.18±0.34	12.19±0.23	12.04±0.71
1	12.13±0.50	11.48±0.01	12.01±0.03	11.53±0.09	12.11±0.09	11.50±0.01
2	12.53±0.00	11.51±0.00	12.57±0.01	11.52±0.07	12.59±0.10	11.51±0.27
3	12.92±0.06	11.53±0.00	12.94±0.56	11.52±0.01	12.97±0.12	11.52±0.18

4	13.56±0.01	11.56±0.00	13.55±0.02	11.54±0.15	13.54±0.01	11.57±0.22
5	13.68±0.06	11.57±0.01	13.61±0.05	11.60±0.04	13.68±0.27	11.64±0.18
6	13.77±0.03	11.70±0.02	13.85±0.08	11.74±0.04	13.91±0.09	11.72±0.20
9	14.17±0.05	12.27±0.76	14.19±0.13	12.37±0.09	14.24±0.34	12.30±0.02
11	14.54±0.01	12.60±0.00	14.55±0.03	12.62±0.04	14.58±0.04	12.54±0.16
13	16.56±0.01	12.88±0.27	15.66±0.19	12.91±0.06	15.62±0.14	12.86±0.11
15	NA	13.16±0.03	15.69±0.02	13.21±0.14	15.70±0.04	13.19±0.04
17	NA	13.25±0.66	15.91±0.02	13.30±0.39	15.94±0.09	13.27±0.08
19	NA	13.51±0.65	16.31±0.02	13.59±0.10	16.35±0.13	13.51±0.14
21	NA	14.38±0.03	16.52±0.36	14.45±0.14	16.48±0.01	14.44±0.09
23	NA	14.80±0.12	16.54±0.08	14.88±0.11	16.63±0.40	14.86±0.07

Days of storage	Spermidine (mg spermidine/kg fish tissue)					
	Treatments€					
	AP	OSP	AUV1	OSUV1	AUV3	OSUV3
0	0.95±0.06	0.93±0.08	0.99±0.22	0.97±0.04	0.98±0.03	0.97±0.03
1	1.13±0.08	0.97±0.02	1.11±0.07	0.98±0.02	1.12±0.08	0.98±0.04
2	1.33±0.13	1.05±0.05	1.36±0.07	1.08±0.06	1.40±0.08	1.09±0.03
3	1.50±0.06	1.22±0.10	1.48±0.08	1.25±0.08	1.46±0.07	1.20±0.07
4	1.58±0.06	1.32±0.08	1.62±0.12	1.37±0.09	1.57±0.06	1.35±0.08
5	1.80±0.01	1.45±0.08	1.83±0.03	1.43±0.08	1.84±0.07	1.46±0.04
6	2.03±0.04	1.64±0.05	2.04±0.08	1.65±0.12	2.05±0.02	1.65±0.01
9	2.26±0.21	1.86±0.01	2.29±0.13	1.83±0.08	2.22±0.09	1.85±0.08
11	2.81±0.05	2.05±0.07	2.82±0.03	2.06±0.05	2.84±0.08	2.07±0.07
13	3.21±0.16	2.41±0.22	3.24±0.07	2.44±0.09	3.28±0.17	2.47±0.06
15	NA	2.66±0.07	3.52±0.13	2.62±0.04	3.58±0.03	2.68±0.10
17	NA	2.90±0.06	3.72±0.14	2.93±0.08	3.76±0.03	2.92±0.04
19	NA	3.27±0.19	3.95±0.07	3.25±0.09	3.92±0.03	3.27±0.08
21	NA	3.76±0.15	4.18±0.19	3.74±0.15	4.20±0.11	3.75±0.07
23	NA	3.78±0.07	4.54±0.09	3.79±0.09	4.64±0.16	3.77±0.08

Results are expressed as means ± standard deviation (n = 2). NA – Not applicable. [€]AP (air packaging); OSP (oxygen scavenger packaging); AUV1 (air packaging + UV-C at 0.102 J/cm^2); OSUV1 (oxygen scavenger packaging + UV-C at 0.102 J/cm^2); AUV3 (air packaging + UV-C at 0.301 J/cm^2); and OSUV3 (oxygen scavenger packaging + UV-C at 0.301 J/cm^2).
